# Supplementary material for: Beyond Bullying, Aggression, Discrimination, and Social Safety: Development of an Integrated Negative Work Behavior Questionnaire (INWBQ)
Source: Int J Environ Res Public Health. 2023 Aug 11;20(16):6564. doi: 10.3390/ijerph20166564 (PMC10454399; doi:10.3390/ijerph20166564)
Supplement: Supplementary file 1 [file ijerph-20-06564-s001.zip › Supplementary Table S1. 44 construct instruments 16 studies on digital NWB, and 32 studies on harm.pdf]

SUPPLEMENTARY TABLE S1. 44 Construct instruments, 16 studies on digital NWB, and 32 studies on harm.

**INSTRUMENTS OF NWB CONSTRUCTS (**tests, questionnaires, scales) **STUDIES**	3. **GOAL**<br>4. **DIGITAL NWB:**<br>Cyber enabled (CE) items[1]<br>Cyber dependent (CD) items	**QUESTIONS ON:**<br>3. **ACTOR TYPE**<br>4. **ACTOR ROLE**<br>**HARM**	**RATING SCALE: ANCHORS**	**DURATION**	**ITEMS**	**RELIABILITY/ CONSISTENCY**
**1. AGGRESSION**						
*Baron Neuman Geddes Scale (BNGS) (Baron et al., 1999)	1. study on the effect of perceived injustice on type A behavior pattern. The grater this type A the greater the engagement in this behavior. Dimensions: expressions of hostility (15), obstructions (10), overt aggression (8).	1. workers<br>2. target<br>Harm: 0 items	1-5: Never to very often	Not specified	33	0.81-0.94
*Indirect Aggression Scale - Target (IAS-T) (Forrest et al., 2005)	1. The development of a psychometric measure of indirect aggression for use in an adult population. 3 Dimensions: Social exclusion (10), malicious humor (9), guilt induction (6).	1. students<br>2. aggressor (IAS-A), target (IAS-T)<br>Harm: 5 double barreled	1-5: Never to regularly	Past 12 months	25	0.81-0.89
*Fox & Stalworth Scale (FSS)(Fox & Stallworth, 2005)	To Explore links between bullying and racism in the US workplace. Dimensions: General (25), racial (7).	1. workers (full time)<br>2. target<br>Harm: 15 items	1-5: Never to extremely often	5 Years	32	0.94<br>0.84
Cyber aggression, dissertation (Weatherbee, 2007)	Construct development, and test of hypothesized relationships of selected variables to	1. coworker, supervisor, customer, person other organization	1-7: Different anchors e.g., agree-disagree,	Last year	116	0.80

Continued SUPPLEMENTARY TABLE S1.

**INSTRUMENTS OF NWB CONSTRUCTS (**tests, questionnaires, scales) **STUDIES**	1. **GOAL**<br>2. **DIGITAL NWB:**<br>Cyber enabled (CE) items[1]<br>Cyber dependent (CD) items	**QUESTIONS ON:**<br>1. **ACTOR TYPE**<br>2. **ACTOR ROLE**<br>**HARM**	**RATING SCALE: ANCHORS**	**DURATION**	**ITEMS**	**RELIABILITY/ CONSISTENCY**
	cyberaggression (by e-mail). Dimensions + items: perceived cyberaggression (25), strains (7), negative affective reactions (19), enacted cyberaggression (35), organization policy (6), employer support (8).<br>2. CE natures	2. target, perpetrator<br>Harm: 7 items	never-all of the time.			
Hospital Aggressive Behavior Scale for coworkers/superiors (HABS-CS) (Waschgler et al., 2013)	1. Brief and manageable instrument for the assessment of aggressive behavior risk.	1. manager, workers<br>2. perpetrator subordinate<br>Harm: 0 items	1-6: Never to daily	Past year	17	0,86
Healthcare-worker's Aggressive Behavior Scale-Users (HABS-U) (Ruiz-Hernández et al., 2016)	1. Measure the prevalence of workplace violence in primary healthcare (PHC) professionals by adapting the HABS-U to establish the frequency of exposure to hostile indicators of clients and to determine which professional group is most exposed. Dimensions: physical, nonphysical.	1. users (clients, visitors, relatives, friends)<br>2. perpetrator<br>Harm: 0 items	1-6: Never, annually, quarterly, monthly, weekly to daily	Past year	10	0.85<br>0.74
2. **BULLYING**						

Continued SUPPLEMENTARY TABLE S1.

**INSTRUMENTS OF NWB CONSTRUCTS (**tests, questionnaires, scales) **STUDIES**	1. **GOAL**<br>2. **DIGITAL NWB:**<br>Cyber enabled (CE) items[1]<br>Cyber dependent (CD) items	**QUESTIONS ON:**<br>1. **ACTOR TYPE**<br>2. **ACTOR ROLE**<br>**HARM**	**RATING SCALE: ANCHORS**	**DURATION**	**ITEMS**	**RELIABILITY/ CONSISTENCY**
* Jóhannsdóttir Ólafsson Scale (JOS)(Jóhannsdóttir & Ólafsson, 2004)	1.Study of bullying, victimization and the coping strategies employed to tackle in Iceland.	1. bankemployees.<br>2. target<br>Harm 1 and 1 double barreled item.	1-5 Never- a few times per week	12 months	18	0.85<br>0.78
*Workplace Bullying Questinnaire- Bullied by Others (WBQ-BO) (Lee & Brotheridge, 2006)	1. Study on counter aggressive/bullying behaviors and certain coping responses on bullying. In turn, if coping with bullying predicted burnout and well-being. Dimensions: belittlement (13), work undermined (7), verbal abuse (7).	1. workers<br>2. target<br>Harm: 0 items	1-5: Not at all to many times a week	6 months	27	0.94
Negative Acts Questionnaire: Revised NAQ-R, (Einarsen et al., 2009)	1. Together with its earlier version, NAQ explores workplace bullying exposure, both in applied and scientific research. Dimensions bullying + items: work related (7), person related (12), physically intimidating (3).	1. coworker<br>2. target<br>Harm: 1 double barreled Item.	1-4: Never, almost never, once a week, more	Past 6 months	22	0.90
Cyber NAQ (Privitera & Campbell, 2009)	1.Investigate the prevalence and methods of face-to-face	1. coworker<br>2. target	1-6: No, yes: very rarely, now	Past 6 months	18	0.94

Continued SUPPLEMENTARY TABLE S1.

**INSTRUMENTS OF NWB CONSTRUCTS (**tests, questionnaires, scales) **STUDIES**	1. **GOAL** 2. **DIGITAL NWB:** Cyber enabled (CE) items[1] Cyber dependent (CD) items	**QUESTIONS ON:** 1. **ACTOR TYPE** 2. **ACTOR ROLE** **HARM**	**RATING SCALE: ANCHORS**	**DURATION**	**ITEMS**	**RELIABILITY/ CONSISTENCY**
	bullying and cyberbullying of males at work. Instrument asks negative acts related to work through different forms of technology. 2. CE all items	Harm:1 item	and then, several times per month, several times per week, and almost daily			
*Escala de Abuso Psicológico Aplicado en el Lugar de Trabajo (EAPA-T) (Escartín et al., 2010)	1 Development and validation of a new measure of workplace bullying instrument.	1. workers 2. target Harm: 1 double barreled item.	1-4: Never to daily	6 months	12	0.89 0.72
*Patchin & Hinduja Cyber bullying scale (PHCS) (Patchin & Hinduja, 2015)	1. Review the essential elements of cyberbullying that distinguish it from other peer-to-peer online interactions and present a cyberbullying scale. Dimensions: offender and victimization scale. 2. CE: 34 items, CD: 2 items.	1. students 2. offender, victim Harm:10 double barreled questions on hurtfulness.	1-4: Never to many times	Past 6 months	36	0.89-0.93 0.93-0.96
Workplace Cyberbullying Measure (WCM) (Farley et al., 2016)	1. Develop a valid and reliable measure to assess cyberbullying across various communication technologies and disparate working populations.	1. coworker 2. target Harm: 1 item	1-5: Never, now and then, at least monthly, at least weekly, daily	Past 6 months	26	0.93

Continued SUPPLEMENTARY TABLE S1.

**INSTRUMENTS OF NWB CONSTRUCTS (**tests, questionnaires, scales) **STUDIES**	**1. GOAL**<br>**2. DIGITAL NWB:**<br>Cyber enabled (CE) items[1]<br>Cyber dependent (CD) items	**QUESTIONS ON:**<br>1. **ACTOR TYPE**<br>**2. ACTOR ROLE**<br>**HARM**	**RATING SCALE: ANCHORS**	**DURATION**	**ITEMS**	**RELIABILITY/ CONSISTENCY**
	2. CE 14 items, 12 face-to face					
Bullying Participants Behavior Questionnaire (BPBQ)(Demaray et al., 2016)	1. To establish reliability and validity of this self-report survey examining various bullying participant role behaviors.	1. students<br>2. bully, victim, assistant, victim, defender victim, outsider.<br>Harm: 0 items	1-5: Never, 1-2 times, 3-5 times, 6 times, 7 or more	Last 30 days	80	0.88<br>0.94
CBQ and CBQ-S (short) (Jönsson et al., 2017)	1. Analyze the reliability and validity of a cyberbullying behavior questionnaire (CBQ) and to analyze the reliability and validity of a short version of a cyberbullying behavior questionnaire (CBQ-S) in working life<br>2. CE:17 items, CD: 3 items	1. coworker<br>2. target<br>Harm:0 items	1-5: Never, now and then, monthly, weekly, daily	Past 6 months	20	0.96<br>Both samples<br><br>Short:<br>0.88
ICA-W (Vranjes et al., 2018)	1. To construct and validate the Inventory of Cyberbullying Acts at Work.<br>2. CE: 8 items, CD: 2 items	1. coworker<br>2. target<br>Harm: 1 item	1-6: Never to always	Past 6 months	10	0.78<br>0.72<br>0.78<br>Full scale:<br>0.81
**Study digital bullying**						

Continued SUPPLEMENTARY TABLE S1.

**INSTRUMENTS OF NWB CONSTRUCTS (**tests, questionnaires, scales) **STUDIES**	1. **GOAL**<br>2. **DIGITAL NWB:**<br>Cyber enabled (CE) items[1]<br>Cyber dependent (CD) items	**QUESTIONS ON:**<br>1. **ACTOR TYPE**<br>2. **ACTOR ROLE**<br>**HARM**	**RATING SCALE: ANCHORS**	**DURATION**	**ITEMS**	**RELIABILITY/ CONSISTENCY**
Internet bullying (Gofin & Avitzour, 2012)	1. To examine the prevalence of traditional and Internet bullying and the personal, family, and school environment characteristics of perpetrators and victims.	1. students 12-14 junior high schools<br>2. perpetrator, victim		hours of internet use a day 1/2–1 h, 1–2 h, more than 2 h		
**Studies harm**<br>Workplace bullying and the risk of cardiovascular disease and depression (Kivimaki et al., 2003)	1.To investigate the association between prolonged bullying and incident depression, and incident cardiovascular disease.	1. hospital employees<br>2. target				
The costs of workplace bullying (Giga e t al., 2008)	1. The use of wide-ranging secondary data sources to extrapolate individual and organizational costs associated with workplace bullying using direct (bullying) and indirect (stress and violence) literature.	Personal costs: Loss of income, additional medical and hospital treatment, costs family. Organizational costs: sickness absence, replacement costs incurred by employee turnover, reduced productivity/performance, knock on effects on witnesses or observers of bullying, premature retirement, grievance and complaints, litigation, and compensation,				

Continued SUPPLEMENTARY TABLE S1.

**INSTRUMENTS OF NWB CONSTRUCTS (**tests, questionnaires, scales) **STUDIES**	1. **GOAL** 2. **DIGITAL NWB:** Cyber enabled (CE) items[1] Cyber dependent (CD) items	**QUESTIONS ON:** 1. **ACTOR TYPE** 2. **ACTOR ROLE** **HARM**	**RATING SCALE: ANCHORS**	**DURATION**	**ITEMS**	**RELIABILITY/ CONSISTENCY**
		organizational intervention.				
Exposure to negative acts at work, psychological stress reactions and physiological stress response (Hogh et al., 2012)	1. To test the association between exposure to negative acts at work, psychological stress-reactions and cortisol secretion and whether some negative acts are more detrimental to health than others.	1. employees of several public and private sectors. 1. target NAQ-R +2 ostracism items, Impact of Event Scale (IES) measured psychological stress (Weiss & Marmar, 1997), Saliva samples to measure cortisol.				
The impact of by standing to workplace bullying on symptoms of depression among women and men in industry in Sweden: an empirical and theoretical longitudinal study (Emdad et al., 2013)	1. To investigate the work environmental risk factors of depressive symptoms among bystanders to bullying in both women and men in four large industrial organizations in Sweden.	1. employees 2. bystanders (witness) Harm: depressive symptoms measured with the Hospital Anxiety and Depression Scale (HAD depression)				
Workplace bullying and sleep difficulties: a 2-year follow-up study (Hansen et al., 2014)	1.To investigate whether being subjected to bullying and witnessing bullying at the	1. workers of public and private workplaces in Denmark 2. target, witness				

Continued SUPPLEMENTARY TABLE S1.

**INSTRUMENTS OF NWB CONSTRUCTS (**tests, questionnaires, scales) **STUDIES**	**1. GOAL** **2. DIGITAL NWB:** Cyber enabled (CE) items[1] Cyber dependent (CD) items	**QUESTIONS ON:** 1. **ACTOR TYPE** **2. ACTOR ROLE** **HARM**	**RATING SCALE: ANCHORS**	**DURATION**	**ITEMS**	**RELIABILITY/ CONSISTENCY**
	workplace was associated with concurrent sleep difficulties.					
Workplace bullying and mental health: A meta-analysis on cross-sectional and longitudinal data. (Verkuil et al., 2015)	1. To examine the relation between workplace bullying and mental health, by pooling the available cross-sectional and longitudinal data (70 samples and a total of170.233 participants), consisting of three categories: (1) symptoms of depression, (2) symptoms of anxiety, and (3) stress- related psychological complaints, such as negative affect and emotional exhaustion.	1. employees 2. targets Harm: widely used scales to measure job induced stress job satisfaction, intention to leave the job / profession, anxiety, depression, PTSD, burnout.				
Workplace bullying in a sample of Italian and Spanish employees and its relationship with job satisfaction, and psychological well-being (Arenas et al., 2015)	1.To examine the prevalence rate of workplace bullying with differential consequences on employees’ job satisfaction and psychological well-being.	1. sample of Italian and Spanish employees 2. target				

Continued SUPPLEMENTARY TABLE S1.

**INSTRUMENTS OF NWB CONSTRUCTS (**tests, questionnaires, scales) **STUDIES**	1. **GOAL**<br>2. **DIGITAL NWB:**<br>Cyber enabled (CE) items[1]<br>Cyber dependent (CD) items	**QUESTIONS ON:**<br>1. **ACTOR TYPE**<br>2. **ACTOR ROLE**<br>**HARM**	**RATING SCALE: ANCHORS**	**DURATION**	**ITEMS**	**RELIABILITY/ CONSISTENCY**
Workplace bullying as a predictor of disability retirement (Nielsen et al., 2017)	1.To determine whether bullying is related to all-cause disability retirement, contributes to disability retirement above high job demands and lack of job control, and to establish gender differences in the relationship.	1. Norwegian employees with disability pension<br>2. target<br>respondents were asked whether they had been subjected to bullying at the workplace during the last 6 months with yes and no. Bullying definition was offered before. Job control measured with QPS (Dallner et al., 2000).				
Workplace bullying and violence as risk factors for type 2 diabetes: a multicohort study and meta-analysis (Xu et al., 2018)	1. Multicohort study to examine if employees exposed to workplace bullying and violence, have an increased risk of type 2 diabetes.	1. Workers: men and women (40– 65 years) free of diabetes in Sweden, Denmark and Finland<br>2. target<br>Bullying and violence were self-reported at baseline + national health and medication records and death registers.				

Continued SUPPLEMENTARY TABLE S1.

**INSTRUMENTS OF NWB CONSTRUCTS (**tests, questionnaires, scales) **STUDIES**	1. **GOAL**<br>2. **DIGITAL NWB:**<br>Cyber enabled (CE) items[1]<br>Cyber dependent (CD) items	**QUESTIONS ON:**<br>1. **ACTOR TYPE**<br>2. **ACTOR ROLE**<br>**HARM**	**RATING SCALE: ANCHORS**	**DURATION**	**ITEMS**	**RELIABILITY/ CONSISTENCY**
**MOBBING**<br>Mobbing with effects on the victim (Leymann, 1996)	1. Research to develop a typology of mobbing activities, subdivided into five dimensions depending on the effects they have on the victim.	1. coworker<br>2. target<br>Harm :5 double barreled items (all)	No anchors	6 months	5	Not identified
Leidse Mobbing Schaal-ll. (LEMS -II) (Hubert & Furda, 1996)	1. Measures the extent of bullying at work and harm.	1. coworker<br>2. target<br>Harm: 12 items	1-5: Never, rarely, at least once a month, at least once a week, daily	Past year	12	0.71-0.88
LIPT-60 scale (Rivera & Abuín, 2003)	1. Translation, validation of Leymann's LIPT-60 questionnaire, extra items added, 6 dimensions: 1. job discrediting,2. hindering progress, 3. communication blockage or blocking of communication,4. covert intimidation,5. overt intimidation and 6 personal discrediting.	1. coworker<br>2. target<br>Harm: 11 items<br>Turkish version health workers (Körükcü et al., 2014)	0-4 Not at all., a little, moderately, very much, extremely.	Past year	60	0.79
**Studies digital mobbing**<br>Cyber-mobbing (Fawzi, 2009)	1. Systematic and differentiated questions on targets and experts	1. coworker<br>2. target				

Continued SUPPLEMENTARY TABLE S1.

**INSTRUMENTS OF NWB CONSTRUCTS (**tests, questionnaires, scales) **STUDIES**	**1. GOAL** **2. DIGITAL NWB:** Cyber enabled (CE) items[1] Cyber dependent (CD) items	**QUESTIONS ON:** 1. **ACTOR TYPE** **2. ACTOR ROLE** **HARM**	**RATING SCALE: ANCHORS**	**DURATION**	**ITEMS**	**RELIABILITY/ CONSISTENCY**
	for an overview on the state-of-the-art research of cyber mobbing.					
Blackmail (Da Silva João & Saldanha Portelada, 2019):	1. Assess the existence, frequency, and intensity of mobbing within the Portuguese nurse population, as well as its impact on their well- being and interpersonal relationships.<br>2. CE	1. people in general<br>2. perpetrator				
**Study harm**<br>Helsemessige aspekter ved mobbing i arbeidslivet: Modererende effekter av sosial støtte og personlighet (Einarsen et al., 1996)	1.To investigate the relationships between exposure to bullying and self-reported health complaints and whether these relationships are moderated by social support and aspects of the victims' personality.	1. workers union members, management<br>2. target<br>The questionnaire included demographic variables, questions on workplace bullying and harassment, several personality scales, health-related measures, and work environment indicators.				
Mobbing at workplace-psychological trauma	1. To identify the features of trauma and analyze the	1. Patiënts<br>2. Target				

Continued SUPPLEMENTARY TABLE S1.

**INSTRUMENTS OF NWB CONSTRUCTS (**tests, questionnaires, scales) **STUDIES**	1. **GOAL** 2. **DIGITAL NWB:** Cyber enabled (CE) items[1] Cyber dependent (CD) items	**QUESTIONS ON:** 1. **ACTOR TYPE** 2. **ACTOR ROLE** **HARM**	**RATING SCALE: ANCHORS**	**DURATION**	**ITEMS**	**RELIABILITY/ CONSISTENCY**
And documentation of psychiatric symptoms (Baran Tatar & Yuksel, 2018)	development of mental problems caused by traumatic experiences in individuals who have been subjected to mobbing at workplace and admitted to psychiatry services.	Trauma Evaluation Form (TIF), Posttraumatic Stress Diagnostic Scale (PDS), Impact of Event Scale- Revised (IES-R) were administered				
**3. HARASSMENT/ DISCRIMINATION**						
*Work Harassment Scale (WHS) (Bjorkqvist et al., 1994)	A series of studies, labeled the "Work Harassment Project," was initiated to investigate the prevalence of work harassment in various types of workplaces in Finland. Aggressor-victim relationships in this study were a part of this.	1. university workers 2. target Harm: 0 items	0-4: Never, very often	6 Months	24	0,95
Ethnic Harassment Experiences scale (EHE) (Schneider et al., 2000)	1. Examines the nature and correlates of the ethnic harassment experiences (i.e., verbal ethnic harassment and exclusion due to ethnicity).	1. coworker 2. target Harm: 0 items	1-5: Never to almost always	Past 24 months	7	0.95 0.90
Generalized Workplace Harassment Questionnaire	1. To measure any negative or hostile workplace interpersonal interactions experienced by an	1. coworker 2. target Harm: 1 item	1-3: Never, once more than twice.	Past year	29	0.92

Continued SUPPLEMENTARY TABLE S1.

**INSTRUMENTS OF NWB CONSTRUCTS (**tests, questionnaires, scales) **STUDIES**	1. **GOAL**<br>2. **DIGITAL NWB:**<br>Cyber enabled (CE) items[1]<br>Cyber dependent (CD) items	**QUESTIONS ON:**<br>1. **ACTOR TYPE**<br>2. **ACTOR ROLE**<br>**HARM**	**RATING SCALE: ANCHORS**	**DURATION**	**ITEMS**	**RELIABILITY/ CONSISTENCY**
(GWHQ) (Rospenda & Richman, 2004)	employee not based on social characteristics such as gender, race ethnic or other social status characteristics that are legally. Five dimensions + items: verbal aggression (9), disrespect (9), isolation/exclusion (5), threats/bribes (3), and physical aggression (3).					
Cyber harassment (Beran & Li, 2005)	1. Measures the form of harassment that occurs using electronic communications such as e-mail and cell phones. Dimensions + items: medium (3), harm (10), combi with vis-à-vis (1), actor type (2). Questions were open ended on type of technology, closed on frequency.<br>2. CE: 3 items:	1. students grade 7-9<br>2. target, perpetrator, bystander<br>Harm: 10 items	1-5: Never, once, twice, a few times, many times, almost every day	Past Year	15	0.88
Gender Experience Questionnaire (SEQ)(Leskinen & Cortina, 2014)	1. Measurement of workplace gender harassment, beyond sexual insults to include hostility targeting one's gender,	1. coworker<br>2. target<br>Harm: 0 items	1-5: Never, once, or twice, sometimes, often, many times	Past year	20	0.85<br>0.90<br>092<br>Full scale

Continued SUPPLEMENTARY TABLE S1.

**INSTRUMENTS OF NWB CONSTRUCTS (**tests, questionnaires, scales) **STUDIES**	**1. GOAL**<br>**2. DIGITAL NWB:**<br>Cyber enabled (CE) items[1]<br>Cyber dependent (CD) items	**QUESTIONS ON:**<br>1. **ACTOR TYPE**<br>**2. ACTOR ROLE**<br>**HARM**	**RATING SCALE: ANCHORS**	**DURATION**	**ITEMS**	**RELIABILITY/ CONSISTENCY**
	gender role (non)conformity, and motherhood status.					
**Study digital discrimination**<br>Discriminating systems: Gender, race, and power in AI (West et al., 2019).	1.Study on algorithms build in AI systems discriminating on gender, race, and power. A diversity crisis in the AI industry and the problems of bias in AI systems are interrelated aspects.<br>2.CD	1. developer, institute, customers<br>2. customer of site as target, institute as perpetrator	analyzing existing IT systems	Year-long pilot review study		
**Studies harm**<br>Harassment as predictor of burnout (Savicki et al., 2003) Local harassment scale on race, religion, gender, national origin (6 items)	To examine the impact of harassment on important aspects of job experience in correctional settings: burnout, commitment to the organization, and perceived stress.	1. correctional officers<br>2. target<br>Harm: locally constructed 6 item scale on harassment based on based on race, religion, gender, or national origin; Maslach Burnout Inventory (Maslach et al., 1997)				
Associations of workplace bullying and harassment with pain (Takaki et al., 2013)	1.To investigate associations of workplace bullying and harassment with headache, stiffness of the neck or	1. workers<br>2. target				

Continued SUPPLEMENTARY TABLE S1.

**INSTRUMENTS OF NWB CONSTRUCTS (**tests, questionnaires, scales) **STUDIES**	**1. GOAL**<br>**2. DIGITAL NWB:**<br>Cyber enabled (CE) items[1]<br>Cyber dependent (CD) items	**QUESTIONS ON:**<br>1. **ACTOR TYPE**<br>**2. ACTOR ROLE**<br>**HARM**	**RATING SCALE: ANCHORS**	**DURATION**	**ITEMS**	**RELIABILITY/ CONSISTENCY**
	shoulders, lumbago, and pain of two or more joints.	NAQ-R + Brief Job Stress Questionnaire(BJSQ, (Shimomitsu et al., 2000)				
Association of sexual harassment and sexual assault with midlife women's mental and physical health (Thurston et al., 2019)	1. To investigate the association of history of sexual harassment and sexual assault with bloodpressure, mood, anxiety, and sleep among midlife women.	1. nonsmoking women aged 40 to 60 years free of clinical cardiovascular disease. Pittsburg, Pennsylvania<br>2. Target<br>Telephone screening Brief Trauma Questionnaire (Koenen et al., 2009), items from the Workplace Sexual Harassment and assault (Breiding et al., 2014) +several physical measurement methods.				
**4. DEVIANCE**<br>Interpersonal and Organizational Deviance Scale (IODS) (Bennett & Robinson, 2000)	1. Research to develop broad, theoretically derived measure(s) of deviant behavior in the workplace.	1. coworker<br>2. perpetrator<br>Harm: 0 items	1-7: Never to once a year, twice a year, several times a	Last Year	19	0.87<br>0.90<br>0.88

Continued SUPPLEMENTARY TABLE S1.

**INSTRUMENTS OF NWB CONSTRUCTS (**tests, questionnaires, scales) **STUDIES**	1. **GOAL** 2. **DIGITAL NWB:** Cyber enabled (CE) items[1] Cyber dependent (CD) items	**QUESTIONS ON:** 1. **ACTOR TYPE** 2. **ACTOR ROLE** **HARM**	**RATING SCALE: ANCHORS**	**DURATION**	**ITEMS**	**RELIABILITY/ CONSISTENCY**
			year, monthly, weekly, daily			
Cyber loafing (Lim, 2002)	1. Study on internet access at work for personal use while pretending to do legitimate work. Measuring engagement in type of cyber loafing if workers experience injustice Justice dimensions + items: distributive (5), procedural (7), interactive (6), forms of cyber loafing (11). 11 CE items of visits on private websites and mail	1. coworker 2. perpetrator Harm: 0 items	A/B 5: Very unfair to very fair C 5: Strongly disagree to strongly agree. D 5: Never to constantly	During working hours	29	0.93
Work related Social Media Questionnaire (WSMQ) (Landers & Callan, 2014)	1. Measure internet surfing during work hours for personal interest, misuse of companies' internet access 2.on CD nature	1. coworker 2. perpetrator	1-5: Strongly disagree- strongly agree.	Last year	9	0.76 0.78
**5. CWB** Counterproductive Work Behavior Checklist -long version (CWB-C) (Fox & Spector, 2002)	1. Measure acts that harm or are intended to harm organizations. They include acts directed toward both organizations and individuals, including	1. coworker 2. perpetrator Harm: 0 items	1-5: Never once or twice, Once or twice per month,	During their job	45	0.87

Continued SUPPLEMENTARY TABLE S1.

**INSTRUMENTS OF NWB CONSTRUCTS (**tests, questionnaires, scales) **STUDIES**	1. **GOAL**<br>2. **DIGITAL NWB:**<br>Cyber enabled (CE) items[1]<br>Cyber dependent (CD) items	**QUESTIONS ON:**<br>1. **ACTOR TYPE**<br>2. **ACTOR ROLE**<br>**HARM**	**RATING SCALE: ANCHORS**	**DURATION**	**ITEMS**	**RELIABILITY/ CONSISTENCY**
	aggression (physical and verbal), sabotage, theft, and withdrawal.		Once or twice per week,<br>Every day.			
**Studies digital CWB**<br>Cyber fraud (Trembly, 2004)	1.Internet communications are not private. 2.CD nature	1. internet users targets	Analyzed court cases.			
Mail and wire fraud (Neese et al., 2005),	1. False statements provided to customers, marketing channel members, and the government. Analysis of federal mail and wire fraud cases related to marketing.<br>2.CE nature	1. marketeers<br>2. perpetrators				
**Study harm**<br>Explaining counterproductive work behaviors among police officers: The indirect effects of job demands are mediated by job burnout and moderated by job control and social support (Smoktunowicz et al., 2015)	1.Part of this study was to investigate the relation of job-demands and CWB on job burnout.	1. police officers<br>2. target				
**6. VIOLENCE**		1. individual/group		Last year	11	

Continued SUPPLEMENTARY TABLE S1.

**INSTRUMENTS OF NWB CONSTRUCTS (**tests, questionnaires, scales) **STUDIES**	1. **GOAL**<br>2. **DIGITAL NWB:**<br>Cyber enabled (CE) items[1]<br>Cyber dependent (CD) items	**QUESTIONS ON:**<br>1. **ACTOR TYPE**<br>2. **ACTOR ROLE**<br>**HARM**	**RATING SCALE: ANCHORS**	**DURATION**	**ITEMS**	**RELIABILITY/ CONSISTENCY**
Violence Research health care: in Brazil, Bulgaria, Lebanon, Portugal, South Africa, Thailand, Australia (Di Martino, 2009)	1.Country studies to identify and address workplace violence in the health sector, to develop sound policies and practical approaches for the prevention and elimination of it.	2. witness<br>Harm: 6 double barreled items	1-5: Not at all, to infrequently (a few times in 12 months), occasionally (a few times each six months), often (a few times each month), frequently (once or more each week),			Not identified
**Studies digital violence** Technology facilitated violence (Henry & Powell, 2016)	1.To examine the scope and limitations of criminal laws for responding to technology-facilitated sexual violence (TFSV) e.g., blackmail, control, coerce, harass, humiliate, objectify, or violate another person.<br>2.CE natures	1. woman in public and private life/ ex partners<br>2. target / perpetrator	Analysis of criminal law responses			
Cyber violence on Twitter (Nagle, 2018)	1. A review of the current literature on social media,	1. students, teachers<br>2. perpetrator, target	Research on Twitter use in			

Continued SUPPLEMENTARY TABLE S1.

**INSTRUMENTS OF NWB CONSTRUCTS (**tests, questionnaires, scales) **STUDIES**	1. **GOAL**<br>2. **DIGITAL NWB:**<br>Cyber enabled (CE) items[1]<br>Cyber dependent (CD) items	**QUESTIONS ON:**<br>1. **ACTOR TYPE**<br>2. **ACTOR ROLE**<br>**HARM**	**RATING SCALE: ANCHORS**	**DURATION**	**ITEMS**	**RELIABILITY/ CONSISTENCY**
	especially Twitter, use in classrooms, to give a multi-disciplinary perspective on issues of cyber-violence and understand the digital tools we use in teacher education.<br>2.CE/CD		teacher education as an educational tool			
**Studies harm**<br>The exploding spark: Workplace violence in an infectious disease hospital-longitudinal study (Magnavita, 2013)	1.Longitudinal study of workplace violence on work-related stress, anxiety, and depression.	1. patients, physicians, nurses<br>2. perpetrator, target<br>Violent Incident Form (Arnetz, 1998) combined with Goldberg scales for anxiety and depression (Goldberg et al., 1988), the Demand Control/ Support Questionnaire for Stress (Karasek, 1979).				
Psychosocial work factors and long sickness absence in Europe (Slany et al., 2014)	1. To investigate workplace violence (physical violence, bullying, and discrimination) relation to long sickness absence.	1. workers<br>2. target<br>Over the past 12 months, at work exposed to: -physical violence -sexual harassment -bullying/harassment -age				

Continued SUPPLEMENTARY TABLE S1.

**INSTRUMENTS OF NWB CONSTRUCTS (**tests, questionnaires, scales) **STUDIES**	1. **GOAL**<br>2. **DIGITAL NWB:**<br>Cyber enabled (CE) items[1]<br>Cyber dependent (CD) items	**QUESTIONS ON:**<br>1. **ACTOR TYPE**<br>2. **ACTOR ROLE**<br>**HARM**	**RATING SCALE: ANCHORS**	**DURATION**	**ITEMS**	**RELIABILITY/ CONSISTENCY**
		discrimination linked to race, ethnic background, or color -discrimination linked to nationality -discrimination on the basis of your sex -discrimination linked to religion -discrimination linked to disability -discrimination linked to sexual orientation At least one situation exposure.				
Criminalizing revenge porn (Citron & Franks, 2014)	1.To argue why criminalization of revenge porn is necessary to protect against the devastating invasion of privacy, sexual privacy, especially the non-consensual publication of sexually graphic images.	Supporting a narrowly and carefully crafted criminal statute can comport with the First Amendment with several laws.				
**7. ABUSE**<br>Abusive Supervision Scale (Tepper, 2000)	1. Drawing on justice theory, to examine the consequences of abusive supervisor behavior.	1. subordinate/supervisor<br>2. subordinate as target, supervisor as perpetrator<br>Harm: 0 items	1-5 Can not remember, seldom, sometimes, moderately often, very often	in the past few months	15	0.90

Continued SUPPLEMENTARY TABLE S1.

**INSTRUMENTS OF NWB CONSTRUCTS (**tests, questionnaires, scales) **STUDIES**	1. **GOAL**<br>2. **DIGITAL NWB:**<br>Cyber enabled (CE) items[1]<br>Cyber dependent (CD) items	**QUESTIONS ON:**<br>1. **ACTOR TYPE**<br>2. **ACTOR ROLE**<br>**HARM**	**RATING SCALE: ANCHORS**	**DURATION**	**ITEMS**	**RELIABILITY/ CONSISTENCY**
**Studies digital abuse**						
Spyware, unauthorized entry into computers (Stafford & Urbaczewski, 2004).	1. indirect infiltration in the form of monitoring programs surreptitiously installed on computers, called spyware, serve to record, and transmit a user's computer uses and behaviors to third parties. Spyware, essentially, is software that asserts control over a user's computer without his/her consent including Adware, Key Loggers, and Trojan Horses.<br>2. CD	1. hackers and users<br>2. e.g., marketeers as perpetrator, customer as target				
Problematic Internet Use (PIU)(Caplan, 2007)	1. Study examined to which extend social anxiety explains results previously attributed to loneliness as a predictor of preference for online social interaction and problematic Internet use.<br>2.CE	1. Internet users<br>2. target, perpetrator				
**Study harm**						

Continued SUPPLEMENTARY TABLE S1.

**INSTRUMENTS OF NWB CONSTRUCTS (**tests, questionnaires, scales) **STUDIES**	1. **GOAL** 2. **DIGITAL NWB:** Cyber enabled (CE) items[1] Cyber dependent (CD) items	**QUESTIONS ON:** 1. **ACTOR TYPE** 2. **ACTOR ROLE** **HARM**	**RATING SCALE: ANCHORS**	**DURATION**	**ITEMS**	**RELIABILITY/ CONSISTENCY**
Abusive supervision and family undermining as displaced aggression (Hoobler & Brass, 2006)	To investigate the results of abusive supervision on subordinates and their family members.	1. subordinates/ family members 2. target				
**8. TERROR**						
**L**eymann Inventory of Psychological Terror (LIPT) (Leymann, 1990)	1.To develop a questionnaire and typography of 45 mobbing actions, 5 dimensions of effects + items: on self-expression (11), on social contact (5), on personal reputation (15), on occupational situation and quality of life (7), on physical health (7).	1. coworker 2. target Harm: 3 double barreled items	1-7: Completely disagree, to strongly disagree, fairly disagree, neither disagree nor agree, fairly agree, strongly agree, completely agree.	Last one year.	45	0.79
Cyberstalking (Every-Palmer et al., 2015). Used FTAC* questionnaire of (James et al., 2013), and (Pathé et al., 2014). *Fixated Threat Assessment Centre (FTAC) is a UK police/mental health unit	1.To investigate the significantly elevated risk of violence, stalking, harassment, and attack to politicians of fixated individuals with untreated serious mental disorders, usually psychosis: in the frequency, nature, and effects. 2. CE	1. Politicians as target (and their family and staff) 2. A small fraction of people with mental illness as harasser divided in approachers and non-approachers. Harm:1 item	Yes/ No: if affirmative further on nature, frequency: 1,2,3-9,>10, location, duration. + additional free text replies.	Current work	42	Not identified
**Study harm**						

Continued SUPPLEMENTARY TABLE S1.

**INSTRUMENTS OF NWB CONSTRUCTS (**tests, questionnaires, scales) **STUDIES**	**1. GOAL**<br>**2. DIGITAL NWB:**<br>Cyber enabled (CE) items[1]<br>Cyber dependent (CD) items	**QUESTIONS ON:**<br>1. **ACTOR TYPE**<br>**2. ACTOR ROLE**<br>**HARM**	**RATING SCALE: ANCHORS**	**DURATION**	**ITEMS**	**RELIABILITY/ CONSISTENCY**
Employee safety perception following workplace terrorism: a longitudinal study (Nissen et al., 2019)	1. To explore longitudinal associations between perceived safety at work among employees exposed to a workplace terrorist attack and their views on security measures and emergency preparedness	1. exposed ministerial employees.<br>2. victim				
**10. INJUSTICE**<br>Combined effect of perceived organizational injustice and perceived politics on deviant behaviors (Khattak et al., 2021)	1. To examine the catalytic impact of perceptions of politics in organizations on the relationship between perceived unfairness and deviant behavior at work. Dimensions +items: distributive (4), procedural (6), interactional (4) justice, perceived organizational politics (15), deviant behavior individual (7), deviant behavior organizational (3) expenses (12).	1. employees<br>2. target-perpetrator-witness role change<br>(Social exchange theory)<br>Harm: 0 items	1-5: strongly disagree to strongly agree.	Current work	48	0.81
**Studies harm**<br>Organizational justice: evidence of a new psychosocial predictor of	1.To examine the justice of decision-making procedures and interpersonal relations as a	1. feminine hospital employees<br>2. target				

Continued SUPPLEMENTARY TABLE S1.

**INSTRUMENTS OF NWB CONSTRUCTS (**tests, questionnaires, scales) **STUDIES**	1. **GOAL** 2. **DIGITAL NWB:** Cyber enabled (CE) items[1] Cyber dependent (CD) items	**QUESTIONS ON:** 1. **ACTOR TYPE** 2. **ACTOR ROLE** **HARM**	**RATING SCALE: ANCHORS**	**DURATION**	**ITEMS**	**RELIABILITY/ CONSISTENCY**
health (Elovainio et al., 2002)	psychosocial predictor of self-rated health, minor psychiatric disorders, and recorded absences due to sickness.					
Justice at work and reduced risk of coronary heart disease among employees (Kivimäki et al., 2005)	1.To investigate the risk of incident coronary heart disease on employees with an intermediate or low level of justice.	1. British civil servants 2. target				
**11. INTERPERSONAL CONFLICT**						
*Interpersonal Workplace events Scale (Not included in this study the 18 positive behavior Items) (IWES) (Keashly et al., 1994)	Study on abusive interpersonal behaviors (hostile verbal, nonverbal, not physical, sexual contact) directed by one or more persons towards another. 3 dimensions: positive (18), abusive (28), physical abusive (2) behaviors.	1. nurses 2. target Harm: 1 double barreled item.	1-5: Rare to always	12 months	48	0.87-0.92
Interpersonal Conflict at Work Scale (ICAWS) (Spector & Jex, 1998)	1. To assesses the frequency with which employees experience arguments and yelling in their interactions with coworkers.	1. workers 2. target Harm: 0 items	1-5: Never to every day	Current work	4	0.74

Continued SUPPLEMENTARY TABLE S1.

**INSTRUMENTS OF NWB CONSTRUCTS (**tests, questionnaires, scales) **STUDIES**	1. **GOAL**<br>2. **DIGITAL NWB:**<br>Cyber enabled (CE) items[1]<br>Cyber dependent (CD) items	**QUESTIONS ON:**<br>1. **ACTOR TYPE**<br>2. **ACTOR ROLE**<br>**HARM**	**RATING SCALE: ANCHORS**	**DURATION**	**ITEMS**	**RELIABILITY/ CONSISTENCY**
**Study digital conflict**<br>Impact of social media on Millennials – a conceptual study (Kavitha & Bhuvaneswari, 2016)	1. Explain the pros and conts of the use of social media on personality development personal conflicts and skills of Millennials with the highest internet use since the last few decades.<br>2.CE natures	1. users of social media<br>2. perpetrators, targets	Secondary data collected from various journals, articles, blogs, publications, and other websites.			
**Studies harm**<br>Workplace conflict resolution and the health of employees in the Swedish and Finnish units of an industrial company (Hyde et al., 2006)	1. To examine the relationship between conflict management in the workplace and self-reported measures of stress, poor general health, exhaustion, and sickness absence due to overstrain or fatigue.	1. non-supervisory employees<br>2. targets<br>The psycho- social work characteristics were measured using the Occupational Stress Questionnaire (OSQ,) (Elo et al., 2003), separate items on conflict management.				
Conflicts at work are associated with a higher risk of cardiovascular disease (Jacob & Kostev, 2017)	1. To analyze the association between workplace conflicts and cardiovascular disorders	1. patients of general practitioners with conflict at work<br>2. target, perpetrator				

Continued SUPPLEMENTARY TABLE S1.

**INSTRUMENTS OF NWB CONSTRUCTS (**tests, questionnaires, scales) **STUDIES**	1. **GOAL**<br>2. **DIGITAL NWB:**<br>Cyber enabled (CE) items[1]<br>Cyber dependent (CD) items	**QUESTIONS ON:**<br>1. **ACTOR TYPE**<br>2. **ACTOR ROLE**<br>**HARM**	**RATING SCALE: ANCHORS**	**DURATION**	**ITEMS**	**RELIABILITY/ CONSISTENCY**
		Measure the incidence of angina pectoris, myocardial infarction, and stroke.				
**12. VICTIMIZATION/ SCAPEGOATING**						
* Perceived Victimization Scale (PVS) ((Aquino & Bradfield, 2000)	1. Study on situational or dispositional characteristics that are likely to produce self-perceptions of victimization. Dimensions: Verbal (12) and covert (6) hostility, manipulation (5), physical hostility (3), sexual harassment (3).	1. governmental agency employees<br>2. Victim<br>Harm: 1 double barreled item.	1-3: Never to more than once	12 Months	10	0.92
Juvenile Victimization Questionnaire Interview (JVQ) (Hamby et al., 2004)	1.Clinical, research, and community settings to help document the true burden of victimization experienced by youth	1. peer, relative<br>2. victim, witness<br>Harm; 5 interview items	Either as a one-year incidence rate or as a frequency of number of incidents in the last year – Yes/No or zero	From birth Until 17 years	34	0.80
Cyberstalking victimization (Reyns et al., 2012)	1. study on pursuit behavior by the internet as a time and spaceless nature. It is repeated	1. intimate, friend, ex friend, acquittance, stranger, coworker,	*0=Non victim, 1=vict1=Intimate, 2=Friend/	Ever	13	Questioned

Continued SUPPLEMENTARY TABLE S1.

**INSTRUMENTS OF NWB CONSTRUCTS (**tests, questionnaires, scales) **STUDIES**	1. **GOAL**<br>2. **DIGITAL NWB:**<br>Cyber enabled (CE) items[1]<br>Cyber dependent (CD) items	**QUESTIONS ON:**<br>1. **ACTOR TYPE**<br>2. **ACTOR ROLE**<br>**HARM**	**RATING SCALE: ANCHORS**	**DURATION**	**ITEMS**	**RELIABILITY/ CONSISTENCY**
	behavior: 4 dimensions + items: unwanted contact, (4) harassment, (2) unwanted sexual advances, and (3) threats of violence or physical harm (4).<br>2. CE	customer, neighbor, relative<br>1. target, perpetrator	Acquaintance, 3=Stranger<br>*1,2,3,4,5, or more persons<br>*Counting incidents			digital response rates.
**13. MICROPOLITICS**<br>Perceptions of Organizational Politics Scale (POPS) (Kacmar & Carlson, 1997)	Introduction of a new scale that purports to measure perceptions of organizational politics. Dimensions + items: general political behavior (2), go along to go ahead (7), pay and promotion policies (6).	1. coworkers, group<br>2. target, witness<br>Harm: 0 items	1-5: Strongly disagree, disagree, neutral, agree, strongly agree.	Context of current work environment	15	0.88
**Study digital politics**<br>Internet politics (Fung et al., 2013)	Bring two opposed perspectives on the influence of digital technologies on politics. Six models: the empowered public sphere, displacement of traditional organizations by new digitally self-organized groups, digitally direct democracy, truth-based advocacy, constituent mobilization, and	1. citizens, interest groups, organizations, governments<br>2. variety of roles	perspectives from scholars of technologies and politics			

Continued SUPPLEMENTARY TABLE S1.

INSTRUMENTS OF NWB CONSTRUCTS (tests, questionnaires, scales) STUDIES	1. GOAL 2. DIGITAL NWB: Cyber enabled (CE) items[1] Cyber dependent (CD) items	QUESTIONS ON: 1. ACTOR TYPE 2. ACTOR ROLE HARM	RATING SCALE: ANCHORS	DURATION	ITEMS	RELIABILITY/ CONSISTENCY
	crowd-sourced social monitoring.					
**14. OSTRACISM** Workplace Ostracism Scale (WOS) (Ferris et al., 2008)	1. Development of a 10-item measure of workplace ostracism.	1. coworker 2. target Harm: 0 items	1-7: Never, once in a while, sometimes, fairly often, often, constantly, always	Past year	10	0.75
**Study digital ostracism** Internet ostracism (Zadro et al., 2004)	1. Compares face to face with two digital forms of social exclusion: 1. exclusion of subjects by a computer, 2. selection by computer of people to exclude subjects. Results show a very primitive and automatic adaptive sensitivity in subjects to even the slightest hint of social exclusion. 2. CE	1. subjects 2. target				
**Study harm** The cost of being ignored: Emotional exhaustion in the	1. To investigate how workplace ostracism both spills over and crosses over to	1. married partners 2. target/ spouse				

Continued SUPPLEMENTARY TABLE S1.

**INSTRUMENTS OF NWB CONSTRUCTS (**tests, questionnaires, scales) **STUDIES**	1. **GOAL**<br>2. **DIGITAL NWB:**<br>Cyber enabled (CE) items[1]<br>Cyber dependent (CD) items	**QUESTIONS ON:**<br>1. **ACTOR TYPE**<br>2. **ACTOR ROLE**<br>**HARM**	**RATING SCALE: ANCHORS**	**DURATION**	**ITEMS**	**RELIABILITY/ CONSISTENCY**
work and family domains. (Thompson et al., 2020)	emotional exhaustion for both the ostracism target and his or her spouse.	Measured with WOS, PANAS (Thompson, 2007), OQ-45.2 (Lambert et al., 1998), MBI ((Maslach et al., 1986), family undermining (Hoobler & Brass, 2006) family emotional exhaustion (Maslach et al., 1986)				
**15. INCIVILITY**<br>Workplace Incivility Scale (WIS) (Cortina et al., 2001)	1.Unidimensional design to assess the frequency of respondent perceptions of disrespectful, rude, or condescending behaviors.	1. superiors, coworkers<br>2. target<br>Harm: 0 items	1-5: Never, once, or twice, sometimes, often, many times	Previous 5 years, also altered to 1 year (Cortina & Magley, 2009), one month (Matthews & Ritter, 2016).	7	0.89
Uncivil Workplace Behavior Questionnaire (UWBQ) (Martin & Hine, 2005)	1.Propose a broader multidimensional construct than the WIS: 4 dimensions +items: hostility (4), privacy invasion	1. managers, collogues.<br>2. target<br>Harm: 0 items	1-5: Never, once, or twice, sometimes, often, many times	From 5 years, altered to 1 year	20	0.65 single factor<br>0.91 four factors.

Continued SUPPLEMENTARY TABLE S1.

**INSTRUMENTS OF NWB CONSTRUCTS (**tests, questionnaires, scales) **STUDIES**	1. **GOAL**<br>2. **DIGITAL NWB:**<br>Cyber enabled (CE) items[1]<br>Cyber dependent (CD) items	**QUESTIONS ON:**<br>1. **ACTOR TYPE**<br>2. **ACTOR ROLE**<br>**HARM**	**RATING SCALE: ANCHORS**	**DURATION**	**ITEMS**	**RELIABILITY/ CONSISTENCY**
	(5), exclusionary behavior (7), gossiping (4).					
Cyber incivility<br>(Lim & Teo, 2009)	1. Examine cyber incivility by mail in the workplace. Compared workplace incivility (15) with outcomes on job satisfaction, (5) quit intentions (3), organizational commitment (9), workplace deviance (20).<br>2. CE	1. coworkers<br>2. target, perpetrator as outcome<br>Harm: 0 items	1-5: Not at all to all the time.	Past year	14	0.95
**Study digital incivility**<br>Cyber-incivility	1. Examine within-person relationships between day-level incivility via work e-mail (cyber incivility) and specifical employee outcomes of detachment at home and distress spillover from one day to the next.<br>2. CE	1. coworkers, supervisors, clients/customers<br>2. target	Yes / No, Number incidents, and Questionnaire Lim &Teo 2009			
**Study harm**<br>Information and communication technology incivility aggression in the workplace: Implications for	1.Examine ICT incivility, related to negative mood state, which in turn may have effect	1. employees<br>2. target, perpetrator<br>Incivility was measured with the 7 points list of Blau & Anderson, separate				

Continued SUPPLEMENTARY TABLE S1.

**INSTRUMENTS OF NWB CONSTRUCTS (**tests, questionnaires, scales) **STUDIES**	1. **GOAL**<br>2. **DIGITAL NWB:**<br>Cyber enabled (CE) items[1]<br>Cyber dependent (CD) items	**QUESTIONS ON:**<br>1. **ACTOR TYPE**<br>2. **ACTOR ROLE**<br>**HARM**	**RATING SCALE: ANCHORS**	**DURATION**	**ITEMS**	**RELIABILITY/ CONSISTENCY**
work and family (Zivnuska et al., 2020)	on attitudes regarding work and family.	instruments for positive mood, psychological distress, job satisfaction, family satisfaction.				
Incivility and bullying in the workplace and nurses' shame responses (Felbinger, 2008)	1. To help the reader of this paper identify disruptive behaviors, describe adverse outcomes of these behaviors, and identify standards that help to create and sustain a healthy work environment.	1. nurses<br>2. victim<br>Local measurement on the network instrument to help identify.				
How employers & co-workers respond to workplace bullying (Namie, 2008)	1.Labor Day 2008 Survey: two separate 400-person respondent groups who visited the WBI website.	1. co-workers, managers<br>2. target, employer<br>Survey report on 11 questions.				
The cost of bad behavior (Porath & Pearson, 2010)	1.To collect data to track the prevalence, types, causes, costs, and cures of incivility at work.	1. managers, workers of 17 industries in US, Canada<br>2. reacting targets, witness<br>Report of people's reactions receiving end of incivility in percentages.				

Continued SUPPLEMENTARY TABLE S1.

**INSTRUMENTS OF NWB CONSTRUCTS (**tests, questionnaires, scales) **STUDIES**	1. **GOAL**<br>2. **DIGITAL NWB:**<br>Cyber enabled (CE) items[1]<br>Cyber dependent (CD) items	**QUESTIONS ON:**<br>1. **ACTOR TYPE**<br>2. **ACTOR ROLE**<br>**HARM**	**RATING SCALE: ANCHORS**	**DURATION**	**ITEMS**	**RELIABILITY/ CONSISTENCY**
Employee adiposity and incivility: Establishing a link and identifying demographic moderators and negative consequences. (Sliter et al., 2012)	1.To explore prevalence of increased adiposity among experienced incivility, burnout and withdrawal on employees in the American workplace	1. self-selected part time workers<br>2. target<br>Self-reported BMI, WIS and demographic variables.				
Mental health expenditures: association with workplace incivility and bullying among hospital patient care workers (Sabbath et al., 2018)	1.To test associations between bullying and health plan claims for mental health diagnoses.	1. hospital workers Boston<br>2. target<br>Measurement with NAQ-R, health care utilization, incurred costs.				
**16. SOCIAL SAFETY**<br>Social Safety Index at work (SVI)(Verschuren, 2012)	1. develop a validated and reliable questionnaire on social safety in various industries. 5 Dimensions +items: characteristics: personal & organizational antecedents (37), incidents: natures, severity, actor types & roles (27), coping: individual, organizational, legal, care, correction (41), consequences, individual,	1. strangers, workers, clients, relatives<br>2. witness, instigator, outsider, collaborator, target, perpetrator<br>Harm: 12 items (organizational), 14 items (individual mental), 7 items (Individual physical)	1-4: Never/ Sometimes/ Usually/ Always	Past year	154	0.56<br>0.90<br>0.87<br>0.91

Continued SUPPLEMENTARY TABLE S1.

**INSTRUMENTS OF NWB CONSTRUCTS (**tests, questionnaires, scales) **STUDIES**	1. **GOAL**<br>2. **DIGITAL NWB:**<br>Cyber enabled (CE) items[1]<br>Cyber dependent (CD) items	**QUESTIONS ON:**<br>1. **ACTOR TYPE**<br>2. **ACTOR ROLE**<br>**HARM**	**RATING SCALE: ANCHORS**	**DURATION**	**ITEMS**	**RELIABILITY/ CONSISTENCY**
	organizational, material, immaterial (26), policy: rules, measures, communication (23).					
Monitor of social safety in primary and secondary education (Scholte et al., 2016; Sijbers et al., 2014)	1. Biennial survey commissioned by the Ministry of Education, Culture and Science in the Netherlands. Dimensions +items: experiences nature violence, feeling of safety, policy, prevention, opportunities for improvement, internal /external care, registration.	1. pupils, staff, management, parents<br>2. perpetrators, target, avoiders<br>Harm: 0 items	open, yes/no, different rating scales and anchors.	12 Months	PO 108 VO 187 (2016)	Not identified
Building digital safety for journalism. (Henrichsen et al., 2015)	1.UNESCO research on personal safety, safety of information, safety of people I work with, safety of sources, safety of family, digital security knowledge and training.<br>2. CE: one question<br>CD: on online platforms and tools	1. online media actors engaged in journalism. in a complex and political climate with sources, collogues, family.<br>2. attacker, target, instigators'<br>Harm: 2 items	Open, yes/no, different rating scales and anchors.	12-18 months<br>Digital use: 0-25%, 26-45%, 46-65%, 66-85%, More than 85%	52	Not identified
**Study digital social safety**						

Continued SUPPLEMENTARY TABLE S1.

**INSTRUMENTS OF NWB CONSTRUCTS (**tests, questionnaires, scales) **STUDIES**	1. **GOAL** 2. **DIGITAL NWB:** Cyber enabled (CE) items[1] Cyber dependent (CD) items	**QUESTIONS ON:** 1. **ACTOR TYPE** 2. **ACTOR ROLE** **HARM**	**RATING SCALE: ANCHORS**	**DURATION**	**ITEMS**	**RELIABILITY/ CONSISTENCY**
Cybercrime, Vandalizing the information society (Furnell, 2002)	This dissertation introduces cyber-dependent crime e.g., like malicious hacking, web defacement, illegal control over IT-systems, malware use. These crimes differ from the cyber-enabled: e.g., online fraud, stalking, harassment.	1. users of digital tools in modern society 2. criminal, target				

*Added instruments in the expert round. Note: unnamed instruments indicated with the abbreviations of the author names.

References Supplementary Table S1:

Aquino, K., & Bradfield, M. (2000). Perceived Victimization in the Workplace: The Role of Situational Factors and Victim Characteristics. *Organization Science*, *11*(5), 525–537. https://doi.org/10.1287/orsc.11.5.525.15205

Arenas, A., Giorgi, G., Montani, F., Mancuso, S., Perez, J. F., Mucci, N., & Arcangeli, G. (2015). Workplace bullying in a sample of Italian and Spanish employees and its relationship with job satisfaction, and psychological well-being. *Frontiers in Psychology*, *6*(DEC), 1–10. https://doi.org/10.3389/fpsyg.2015.01912

Arnetz, J. E. (1998). The Violent Incident Form (VIF): A practical instrument for the registration of violent incidents in the health care workplace. *Work & Stress*, *12*(1), 17–28. https://doi.org/10.1080/02678379808256846

Baran Tatar, Z., & Yuksel, S. (2018). Mobbing at workplace - Psychological trauma and documentation of psychiatric symptoms. *Archives of Neuropsychiatry*, *56*(1), 57–62. https://doi.org/10.29399/npa.22924

Baron, R. A., Neuman, J. H., & Geddes, D. (1999). Social and personal determinants of workplace aggression: Evidence for the impact of perceived injustice and the Type A Behavior Pattern. *Aggressive Behavior*, *25*(4), 281–296. https://doi.org/10.1002/(SICI)1098-2337(1999)25:4<281::AID-AB4>3.0.CO;2-J

Bennett, R. J., & Robinson, S. L. (2000). Development of a measure of workplace deviance. *Journal of Applied Psychology*, *85*(3), 349–360. https://doi.org/10.1037/0021-9010.85.3.349

Continued SUPPLEMENTARY TABLE S1.

**INSTRUMENTS OF NWB CONSTRUCTS** (tests, questionnaires, scales) **STUDIES**	1. **GOAL** 2. **DIGITAL NWB:** Cyber enabled (CE) items[1] Cyber dependent (CD) items	**QUESTIONS ON:** 1. **ACTOR TYPE** 2. **ACTOR ROLE** **HARM**	**RATING SCALE: ANCHORS**	**DURATION**	**ITEMS**	**RELIABILITY/ CONSISTENCY**

Beran, T., & Li, Q. (2005). Cyber-Harassment: A Study of a New Method for an Old Behavior. *Journal of Educational Computing Research*, *32*(3), 265–277. https://doi.org/10.2190/8YQM-B04H-PG4D-BLLH

Bjorkqvist, K., Osterman, K., & Hielt-Bdck, M. (1994). Aggression Among University Employees. *Agressive Behaviour*, *20*, 173–184. https://doi.org/10.1002/1098-2337(1994)20

Breiding, M. J., Smith, S. G., Basile, K. C., Walters, M. L., Chen, J., & Merrick, M. T. (2014). Prevalence and characteristics of sexual violence, stalking, and intimate partner violence victimization—national intimate partner and sexual violence survey, United States, 2011. *MMWR Surveill Summ.*, *63*(8), 1–18.

Caplan, S. E. (2007). Relations among loneliness, social anxiety, and problematic internet use. *CyberPsychology & Behavior*, *10*(2), 234–242. https://doi.org/10.1089/cpb.2006.9963

Citron, D. K., & Franks, M. A. (2014). Criminalizing revenge porn. *Wake Forest Law Review 345*, *2014*. https://scholarship.law.bu.edu/faculty_scholarship/643

Cortina, L. M., Magley, V. J., Williams, J. H., & Langhout, R. D. (2001). Incivility in the workplace: Incidence and impact. *Journal of Occupational Health Psychology*, *6*(1), 64–80. https://doi.org/10.1037/1076-8998.6.1.64

Da Silva João, A. L., & Saldanha Portelada, A. F. (2019). Mobbing and its impact on interpersonal relationships at the workplace. *Journal of Interpersonal Violence*, *34*(13), 2797–2812. https://doi.org/10.1177/0886260516662850

Dallner, M., Elo, A., Gamberale, L. F., & Al, E. (2000). *Validation of the General Nordic Questionnaire (QPSNordic) for psychological and social factors at work.* Nordic Council of Ministers.

Demaray, M. K., Summers, K. H., Jenkins, L. N., & Becker, L. D. (2016). Bullying Participant Behaviors Questionnaire (BPBQ): Establishing a Reliable and Valid Measure. *Journal of School Violence*, *15*(2), 158–188. https://doi.org/10.1080/15388220.2014.964801

Di Martino, V. (2009). *Workplace violence in the health sector Country case studies Brazil, Bulgaria, Lebanon, Portugal, South Africa, Thailand and an additional Australian study. Synthesis report.* https://www.who.int/violence_injury_prevention/violence/activities/workplace/WVsynthesisreport.pdf

Einarsen, S, Hoel, H., & Notelaers, G. (2009). Measuring exposure to bullying and harassment at work: Validity, factor structure and psychometric properties of the Negative Acts Questionnaire-Revised. *Work & Stress*, *23*(1), 24–44. https://doi.org/10.1080/02678370902815673

Einarsen, Ståle, Raknes, B. I., Matthiesen, S. B., & Hellesøy, O. H. (1996). Helsemessige aspekter ved mobbing i arbeidslivet: Modererende

Continued SUPPLEMENTARY TABLE S1.

**INSTRUMENTS OF NWB CONSTRUCTS (**tests, questionnaires, scales) **STUDIES**	1. **GOAL** 2. **DIGITAL NWB:** Cyber enabled (CE) items[1] Cyber dependent (CD) items	**QUESTIONS ON:** 1. **ACTOR TYPE** 2. **ACTOR ROLE** **HARM**	**RATING SCALE: ANCHORS**	**DURATION**	**ITEMS**	**RELIABILITY/ CONSISTENCY**

effekter av sosial støtte og personlighet. *Nordisk Psykologi*, *48*(2), 116–137. https://doi.org/10.1080/00291463.1996.11863874

Elo, A.-L., Leppänen, A., & Jahkola, A. (2003). Validity of a single-item measure of stress symptoms. *Scandinavian Journal of Work, Environment & Health*, *29*(6), 444–451. https://doi.org/10.5271/sjweh.752

Elovainio, M., Kivimaki, M., & Vahtera, J. (2002). Organizational justice: evidence of a new psychosocial predictor of health. *American Journal of Public Health*, *92*, 105–108. https://ajph.aphapublications.org/doi/pdfplus/10.2105/AJPH.92.1.105

Emdad, R., Alipour, A., Hagberg, J., & Jensen, I. B. (2013). The impact of bystanding to workplace bullying on symptoms of depression among women and men in industry in Sweden: an empirical and theoretical longitudinal study. *International Archives of Occupational and Environmental Health*, *86*(6), 709–716. https://doi.org/10.1007/s00420-012-0813-1

Escartín, J., Rodríguez-Carballeira, Á., Gómez-Benito, J., & Zapf, D. (2010). Development and validation of the workplace bullying scale EAPA-T. *International Journal of Clinical and Health Psychology*, *10*(3), 519–539. http://www.redalyc.org/articulo.oa?id=33714079008

Every-Palmer, S., Barry-Walsh, J., & Pathé, M. (2015). Harassment, stalking, threats and attacks targeting New Zealand politicians: A mental health issue. *Australian & New Zealand Journal of Psychiatry*, *49*(7), 634–641. https://doi.org/10.1177/0004867415583700

Farley, S., Coyne, I., Axtell, C., & Sprigg, C. (2016). Design, development and validation of a workplace cyberbullying measure, the WCM. *Work & Stress*, *30*(4), 293–317. https://doi.org/10.1080/02678373.2016.1255998

Fawzi, N. (2009). Cyber-mobbing: Ursachen und Auswirkungen von Mobbing im Internet (Cyber-mobbing: Causes and effects of mobbing on the internet). In K. Beck, J. Höflich, K. Kamps, F. Krotz, W. Schweiger, W. Wirth, & Band (Eds.), *Nomos Internet Research* (Band 37). Edition Reinhard Fischer.

Felblinger, D. M. (2008). Incivility and bullying in the workplace and nurses' shame responses. *Journal of Obstetric, Gynecologic & Neonatal Nursing*, *37*(2), 234–242. https://doi.org/10.1111/j.1552-6909.2008.00227.x

Ferris, D. L., Brown, D. J., Berry, J. W., & Lian, H. (2008). The development and validation of the Workplace Ostracism Scale. *Journal of Applied Psychology*, *93*(6), 1348–1366. https://doi.org/10.1037/a0012743

Forrest, S., Eatough, V., & Shevlin, M. (2005). Measuring adult indirect aggression: The development and psychometric assessment of the indirect aggression scales. *Aggressive Behavior*, *31*(1), 84–97. https://doi.org/10.1002/ab.20074

Fox, S., & Spector, P. E. (2002). *cwb-c-45-english*.

Fox, S., & Stallworth, L. E. (2005). Racial/ethnic bullying: Exploring links between bullying and racism in the US workplace. *Journal of Vocational Behavior*, *66*(3), 438–456. https://doi.org/10.1016/j.jvb.2004.01.002

Continued SUPPLEMENTARY TABLE S1.

**INSTRUMENTS OF NWB CONSTRUCTS (**tests, questionnaires, scales) **STUDIES**	1. **GOAL**<br>2. **DIGITAL NWB:**<br>Cyber enabled (CE) items[1]<br>Cyber dependent (CD) items	**QUESTIONS ON:**<br>1. **ACTOR TYPE**<br>2. **ACTOR ROLE**<br>**HARM**	**RATING SCALE: ANCHORS**	**DURATION**	**ITEMS**	**RELIABILITY/ CONSISTENCY**

Fung, A., Russon Gilman, H., & Shkabatur, J. (2013). Six models for the internet + politics. *International Studies Review*, *15*(1), 30–47. https://doi.org/10.1111/misr.12028

Furnell, S. (2002). *Cybercrime: Vandalizing the information society*. Addison-Wesley.

Giga, S. I., Hoel, H., & Lewis, D. (2008). *The costs of workplace bullying*. https://www.researchgate.net/publication/260246863_The_Costs_of_Workplace_Bullying

Gofin, R., & Avitzour, M. (2012). Traditional Versus Internet Bullying in Junior High School Students. *Maternal and Child Health Journal*, *16*(8), 1625–1635. https://doi.org/10.1007/s10995-012-0989-8

Goldberg, D., Bridges, K., Duncan-Jones, P., & Grayson, D. (1988). Detecting anxiety and depression in general medical settings. *BMJ*, *297*(6653), 897–899. https://doi.org/10.1136/bmj.297.6653.897

Hamby, S. L., Finkelhor, D., Ormrod, R., Turner, H., Hamby, S. L., Finkelhor, D., Ormrod, R., & Turner, H. (2004). *The Juvenile Victimization Questionnaire (JVQ): Administration and scoring maual* (Number March). Crimes Against Children Research Centre.

Hansen, Å. M., Hogh, A., Garde, A. H., & Persson, R. (2014). Workplace bullying and sleep difficulties: a 2-year follow-up study. *International Archives of Occupational and Environmental Health*, *87*(3), 285–294. https://doi.org/10.1007/s00420-013-0860-2

Henrichsen, J. R., Betz, M., & Lisosky, J. M. (2015). Building Digital Safety for Journalism: a Survey of Selected Issues. In *Unesco Series on Internet Freedom*. http://unesdoc.unesco.org/images/0023/002323/232358e.pdf

Henry, N., & Powell, A. (2016). Sexual violence in the digital age. *Social & Legal Studies*, *25*(4), 397–418. https://doi.org/10.1177/0964663915624273

Hogh, A., Hansen, Å. M., Mikkelsen, E. G., & Persson, R. (2012). Exposure to negative acts at work, psychological stress reactions and physiological stress response. *Journal of Psychosomatic Research*, *73*(1), 47–52. https://doi.org/10.1016/j.jpsychores.2012.04.004

Hoobler, J. M., & Brass, D. J. (2006). Abusive supervision and family undermining as displaced aggression. *Journal of Applied Psychology*, *91*(5), 1125–1133. https://doi.org/10.1037/0021-9010.91.5.1125

Hubert, A. B., & Furda, J. (1996). *Leidse Mobbing Schaal-II (LEMS-II; geïndividualiseerde versie (Leiden Mobbing Scale-II (LEMS-II individualised version)*. Leiden University.

Hyde, M., Jappinen, P., Theorell, T., & Oxenstierna, G. (2006). Workplace conflict resolution and the health of employees in the Swedish and Finnish units of an industrial company. *Social Science & Medicine*, *63*(8), 2218–2227. https://doi.org/10.1016/j.socscimed.2006.05.002

Jacob, L., & Kostev, K. (2017). Conflicts at work are associated with a higher risk of cardiovascular disease. *GMS German Medical Science*, *15*,

Continued SUPPLEMENTARY TABLE S1.

**INSTRUMENTS OF NWB CONSTRUCTS (**tests, questionnaires, scales) **STUDIES**	**1. GOAL** **2. DIGITAL NWB:** Cyber enabled (CE) items[1] Cyber dependent (CD) items	**QUESTIONS ON:** 1. **ACTOR TYPE** **2. ACTOR ROLE** **HARM**	**RATING SCALE: ANCHORS**	**DURATION**	**ITEMS**	**RELIABILITY/ CONSISTENCY**

1–8. https://doi.org/10.3205/000249

James, D. V., Farnham, F. R., & Wilson, S. P. (2013). The fixated threat assessment centre. In J. . Meloy & J. Hoffmann (Eds.), *International Handbook of Threat Assessmen* (pp. 299–320). Oxford University Press. https://fliphtml5.com/pkir/qlmx

Jóhannsdóttir, H. L., & Ólafsson, R. F. (2004). Coping with bullying in the workplace: the effect of gender, age and type of bullying. *British Journal of Guidance & Counselling*, *32*(3), 319–333. https://doi.org/10.1080/03069880410001723549

Jönsson, S., Muhonen, T., Forssell, R. C., & Bäckström, M. (2017). Assessing Exposure to Bullying through Digital Devices in Working Life: Two Versions of a Cyberbullying Questionnaire (CBQ). *Psychology*, *08*(03), 477–494. https://doi.org/10.4236/psych.2017.83030

Kacmar, K. M., & Carlson, D. S. (1997). Further validation of the perceptions of politics scale (POPS): A multiple sample investigation. *Journal of Management*, *23*(5), 627–658. https://doi.org/10.1177/0013164491511019

Karasek, R. A. (1979). Job demands, job decision latitude, and mental strain. Implication for job redesign. *Administrative Science Quarterly, 24*(2), 285–308. https://www.jstor.org/stable/2392498

Kavitha, S., & Bhuvaneswari, R. (2016). Impact of social media on millenials- a conceptual study. *Journal of Management Sciences and Technology*, *4*(1), 80–86. https://www.apeejay.edu/aitsm/journal/docs/issue-oct-2016/ajmst040108.pdf

Keashly, L., Trott, V., & MacLean, L. M. (1994). Abusive Behavior In the workplace: a_prel.pdf. *Violence and Victims*, *9*(4), 341–357. https://d1wqtxts1xzle7.cloudfront.net/53005802/Abusive_behavior_in_the_workplace_a_prel20170505-3112-y427a7-libre.pdf?1493978131=&response-content-disposition=inline%3B+filename%3DAbusive_behavior_in_the_workplace_a_prel.pdf&Expires=1680987381&Signature=D

Khattak, M. N., Zolin, R., & Muhammad, N. (2021). The combined effect of perceived organizational injustice and perceived politics on deviant behaviors. *International Journal of Conflict Management*, *32*(1), 62–87. https://doi.org/10.1108/IJCMA-12-2019-0220

Kivimäki, M., Ferrie, J. E., Brunner, E., Head, J., Shipley, M. J., Vahtera, J., & Marmot, M. G. (2005). Justice at Work and Reduced Risk of Coronary Heart Disease Among Employees. *Archives of Internal Medicine*, *165*(19), 2245. https://doi.org/10.1001/archinte.165.19.2245

Kivimaki, M., Virtanen, M., Vartia, M., Elovainio, M., Vahtera, J., & .... L. K.-J. (2003). Workplace bullying and the risk of cardiovascular disease and depression. *Occupational & Environmental Medicine*, *60*, 779–783. https://oem.bmj.com/content/60/10/779.short

Koenen, K. C., De Vivo, I., Rich-Edwards, J., Smoller, J. W., Wright, R. J., & Purcell, S. M. (2009). Protocol for investigating genetic determinants of posttraumatic stress disorder in women from the Nurses' Health Study II. *BMC Psychiatry*, *9*(1), 29.

Continued SUPPLEMENTARY TABLE S1.

**INSTRUMENTS OF NWB CONSTRUCTS (**tests, questionnaires, scales) **STUDIES**	1. **GOAL** 2. **DIGITAL NWB:** Cyber enabled (CE) items[1] Cyber dependent (CD) items	**QUESTIONS ON:** 1. **ACTOR TYPE** 2. **ACTOR ROLE** **HARM**	**RATING SCALE: ANCHORS**	**DURATION**	**ITEMS**	**RELIABILITY/ CONSISTENCY**

https://doi.org/10.1186/1471-244X-9-29

Körükcü, Ö., Bulut, O., Tuzcu, A., Bayram, Z., & Öztürk Türkmen, H. (2014). An adaptation of Leymann Inventory of Psychological Terror to health sciences programs in Turkey. *Anatolian Journal of Psychiatry*, *15*(4), 335–343. https://doi.org/10.5455/apd.42654

Lambert, M. J., Finch, A. M., Okiishi, J., Burlingame, G. M., McKelvey, C., & Reisinger, C. W. (. (1998). *Administration and scoring manual for the OQ-10.2: An adult outcome questionnaire for screening individuals and population outcome monitoring.* Stevenson MD: American Professional Credentialing Services. https://journals.sagepub.com/doi/abs/10.2466/02.08.PR0.112.3.689-693

Landers, R. N., & Callan, R. C. (2014). Validation of the beneficial and harmful work-related social media behavioral taxonomies. *Social Science Computer Review*, *32*(5), 628–646. https://doi.org/10.1177/0894439314524891

Lee, R. T., & Brotheridge, C. M. (2006). When prey turns predatory: Workplace bullying as a predictor of counteraggression/bullying, coping, and well-being. *European Journal of Work and Organizational Psychology*, *15*(3), 352–377. https://doi.org/10.1080/13594320600636531

Leskinen, E. A., & Cortina, L. M. (2014). Dimensions of disrespect: Mapping and measuring gender harassment in organizations. *Psychology of Women Quarterly*, *38*(1), 107–123. https://doi.org/10.1177/0361684313496549

Leymann, H. (1990). Mobbing and psychological terror at workplaces. *Violence and Victims*, *5*, 119–126. https://www.mobbingportal.com/LeymannV&V1990(3).pdf

Leymann, H. (1996). The content and development of mobbing at work. *European Journal of Work and Organizational Psychology*, *5*(2), 165–184. https://doi.org/10.1080/13594329608414853

Lim, V. K. (2002). The IT way of loafing on the job: cyberloafing, neutralizing and organizational justice. *Journal of Organizational Behavior*, *23*(5), 675–694. https://doi.org/10.1002/job.161

Magnavita, N. (2013). The exploding spark: Workplace violence in an infectious disease hospital- longitudinal study. *BioMed Research International*, 1–9. https://doi.org/10.1155/2013/316358

Martin, R. J., & Hine, D. W. (2005). Development and validation of the Uncivil Workplace Behavior Questionnaire. *Journal of Occupational Health Psychology*, *10*(4), 477–490. https://doi.org/10.1037/1076-8998.10.4.477

Maslach, C., Jackson, S. E., & Head, M. P. (1986). *Maslach Burnout Inventory.* Consulting Psychologists Press.

Maslach, C., Jackson, S. E., & Leiter, M. P. (1997). Maslach Burnout Inventory: In C. P. Zalaquett & R. J. Wood (Eds.), *Evaluating stress: A book of resources* (3rd ed., pp. 191–218). Scarecrow Education.

Nagle, J. (2018). Twitter, cyber-violence, and the need for a critical social media literacy in teacher education: A review of the literature.

Continued SUPPLEMENTARY TABLE S1.

**INSTRUMENTS OF NWB CONSTRUCTS (**tests, questionnaires, scales) **STUDIES**	1. **GOAL** 2. **DIGITAL NWB:** Cyber enabled (CE) items[1] Cyber dependent (CD) items	**QUESTIONS ON:** 1. **ACTOR TYPE** 2. **ACTOR ROLE HARM**	**RATING SCALE: ANCHORS**	**DURATION**	**ITEMS**	**RELIABILITY/ CONSISTENCY**

Teaching and Teacher Education, *76*(September), 86–94. https://doi.org/10.1016/j.tate.2018.08.014

Namie, G. (2008). *How employers pay for bullying*. http://bullyinginstitute.org/education/bbstudies/econ.html

Neese, W. T., Ferrell, L., & Ferrell, O. C. (2005). An analysis of federal mail and wire fraud cases related to marketing. *Journal of Business Research*, *58*(7), 910–918. https://doi.org/10.1016/j.jbusres.2004.01.010

Nielsen, M. ., Emberland, J. S., & Knardahl, S. (2017). Workplace bullying as a predictor of disability retirement. *Journal of Occupational and Environmental Medicine*, *59*(7), 609–614. https://doi.org/10.1097/JOM.0000000000001026

Nissen, A., Hansen, M. B., Nielsen, M. ., Knardahl, S., & Heir, T. (2019). Employee safety perception following workplace terrorism: a longitudinal study. *European Journal of Psychotraumatology*, *10*(1), 1478584. https://doi.org/10.1080/20008198.2018.1478584

Patchin, J. W., & Hinduja, S. (2015). Measuring cyberbullying: Implications for research. *Aggression and Violent Behavior*, *23*, 69–74. https://doi.org/10.1016/j.avb.2015.05.013

Pathé, M., Phillips, J., Perdacher, E., & Heffernan, E. (2014). The Harassment of Queensland Members of Parliament: A Mental Health Concern. *Psychiatry, Psychology and Law*, *21*(4), 577–584. https://doi.org/10.1080/13218719.2013.858388

Porath, C. L., & Pearson, C. M. (2010). The cost of bad behavior. *Organizational Dynamics*, *39*(1), 64–71. https://doi.org/10.1016/j.orgdyn.2009.10.006

Privitera, C., & Campbell, M. . (2009). Cyberbullying: The new face of workplace bullying? *Cyber Psychology and Behavior*, *12*, 395-400. https://doi.org/org/10.1089/cpb.2009.0025 PDF/EPUB

Reyns, B. W., Henson, B., & Fisher, B. S. (2012). Stalking in the twilight zone: Extent of cyberstalking victimization and offending among college students. *Deviant Behavior*, *33*(1), 1–25. https://doi.org/10.1080/01639625.2010.538364

Rivera, J., & Abuín, M. (2003). Cuestionario de estrategias de acoso en el trabajo: El LIPT-60 (Leymann Inventory of Pychological Terrorization) en version espa- ñola [Questionnaire of etrategies of harrassment at the workplace: El LIPT-60 (Leymann Inventory of Pychological Terrorizatio. *Psiquis*, *24*(2), 59–69. https://www.academia.edu/33980625/Cuestionario_de_estrategias_de_acoso_psicologico_Leymann_Inventory_of_Psychological_Terroriza tion

Rospenda, K. M., & Richman, J. A. (2004). The factor structure of generalized workplace harassment. *Violence and Victims*, *19*(2), 221–238. https://doi.org/10.1891/088667004780927963

Ruiz-Hernández, J. A., López-García, C., Llor-Esteban, B., Galián-Muñoz, I., & Benavente-Reche, A. P. (2016). Evaluation of the users violence

Continued SUPPLEMENTARY TABLE S1.

**INSTRUMENTS OF NWB CONSTRUCTS (**tests, questionnaires, scales) **STUDIES**	1. **GOAL** 2. **DIGITAL NWB:** Cyber enabled (CE) items[1] Cyber dependent (CD) items	**QUESTIONS ON:** 1. **ACTOR TYPE** 2. **ACTOR ROLE** **HARM**	**RATING SCALE: ANCHORS**	**DURATION**	**ITEMS**	**RELIABILITY/ CONSISTENCY**

in primary health care: Adaptation of an instrument. *International Journal of Clinical and Health Psychology*, *16*(3), 295–305. https://doi.org/10.1016/j.ijchp.2016.06.001

Sabbath, E. L., Williams, J. A. R., Boden, L. I., Tempesti, T., Wagner, G. R., Hopcia, K., Hashimoto, D., & Sorensen, G. (2018). Mental health expenditures: Association with workplace incivility and bullying among hospital patient care workers. *Journal of Occupational and Environmental Medicine*, *60*(8), 737–742. https://doi.org/10.1097/JOM.0000000000001322

Savicki, V., Cooley, E., & Gjesvold, J. (2003). Harassment as a Predictor of Job Burnout in Correctional Officers. *Criminal Justice and Behavior*, *30*(5), 602–619. https://doi.org/10.1177/0093854803254494

Schneider, K. T., Hitlan, R. T., & Radhakrishnan, P. (2000). An examination of the nature and correlates of ethnic harassment experiences in multiple contexts. *Journal of Applied Psychology*, *85*(1), 3–12. https://doi.org/10.1037/0021-9010.85.1.3

Scholte, R., Nelen, W., De Wit, W., & Kroes, G. (2016). *Sociale veiligheid in en rond scholen [Social safety in and around schools] 2010-2016* (Number december). https://www.rijksoverheid.nl/documenten/rapporten/2016/12/23/sociale-veiligheid-in-en-rond-scholen

Shimomitsu, T., Haratani, T., Iwata, N., & Nakamura, K. (2000). The Final Development of the Brief Job Stress Questionnaire Mainly Used for Assessment of the Individuals. In M. Kato (Ed.), *A Research Report Relating to Stress in the Workplace and its Impact on Workers' Health* (ored Grant, pp. 126–164). Ministry of Labour.

Sijbers, R., De Wit, W., Fettelaar, D., & Mooij, T. (2014). *Sociale veiligheid in en rond scholen. Primair (speciaal) onderwijs 2010-2014; Voortgezet (speciaal) onderwijs 2006-2014. [Social safety in and around schools. Primary (special) education 2010-2014; Secundairy (special) education 2006-2014]*. https://www.researchgate.net/publication/285926152%0ASijbers,

Slany, C., Schütte, S., Chastang, J.-F., Parent-Thirion, A., Vermeylen, G., & Niedhammer, I. (2014). Psychosocial work factors and long sickness absence in Europe. *International Journal of Occupational and Environmental Health*, *20*(1), 16–25. https://doi.org/10.1179/2049396713Y.0000000048

Sliter, K. A., Sliter, M. T., Withrow, S. A., & Jex, S. M. (2012). Employee adiposity and incivility: Establishing a link and identifying demographic moderators and negative consequences. *Journal of Occupational Health Psychology*, *17*(4), 409–424. https://doi.org/10.1037/a0029862

Smoktunowicz, E., Baka, L., Cieslak, R., Nichols, C. F., Benight, C. C., & Luszczynska, A. (2015). Explaining counterproductive work behaviors among police officers: The indirect effects of job demands are mediated by job burnout and moderated by job control and social support. *Human Performance*, *28*(4), 332–350. https://doi.org/10.1080/08959285.2015.1021045

Continued SUPPLEMENTARY TABLE S1.

**INSTRUMENTS OF NWB CONSTRUCTS (**tests, questionnaires, scales) **STUDIES**	1. **GOAL** 2. **DIGITAL NWB:** Cyber enabled (CE) items[1] Cyber dependent (CD) items	**QUESTIONS ON:** 1. **ACTOR TYPE** 2. **ACTOR ROLE** **HARM**	**RATING SCALE: ANCHORS**	**DURATION**	**ITEMS**	**RELIABILITY/ CONSISTENCY**

Spector, P. E., & Jex, S. M. (1998). Development of four self-report measures of job stressors and strain: Interpersonal conflict at work scale, organizational constraints scale, quantitative workload inventory, and physical symptoms inventory. *Journal of Occupational Health Psychology*, *3*(4), 356–367. https://psycnet.apa.org/buy/1998-12418-005

Stafford, T. F., & Urbaczewski, A. (2004). Spyware: The ghost in the machine. *Communications of the Association for Information Systems*, *14*, 291–306. http://130.18.86.27/faculty/warkentin/SecurityPapers/Merrill/StaffordUrbaczewski2004_CAIS14_Spyware.pdf

Takaki, J., Taniguchi, T., & Hirokawa, K. (2013). Associations of workplace bullying and harassment with pain. *International Journal of Environmental Research and Public Health*, *10*(10), 4560–4570. https://doi.org/10.3390/ijerph10104560

Tepper, B. J. (2000). Consequences of Abusive Supervision. *Academy of Management Journal*, *43*(2), 178–190. https://doi.org/10.5465/1556375

Thompson, E. R. (2007). Development and Validation of an Internationally Reliable Short-Form of the Positive and Negative Affect Schedule (PANAS). *Journal of Cross-Cultural Psychology*, *38*(2), 227–242. https://doi.org/10.1177/0022022106297301

Thompson, M. J., Carlson, D. S., Kacmar, K. M., & Vogel, R. M. (2020). The cost of being ignored: Emotional exhaustion in the work and family domains. *Journal of Applied Psychology*, *105*(2), 186–195. https://doi.org/10.1037/apl0000433

Thurston, R. C., Chang, Y., Matthews, K. A., von Känel, R., & Koenen, K. (2019). Association of sexual harassment and sexual assault with midlife women's mental and physical health. *JAMA Internal Medicine*, *179*(1), 48. https://doi.org/10.1001/jamainternmed.2018.4886

Trembly, A. C. (2004). E-mails nail wrongdoers. *National Underwriter, P&C*, *108*(41), 5. https://doi.org/10.1002/imhj.20015

Verkuil, B., Atasayi, S., & Molendijk, M. L. (2015). Workplace bullying and mental health : A meta- analysis on cross-sectional and longitudinal data. *PLoS ONE*, *10*(8), 1–17. https://doi.org/10.1371/journal. pone0135225

Verschuren, C. M. (2012). *Handleiding Sociale veiligheidsindex: Sociale veiligheid binnen de werkomgeving (Manual Social Safetyindex: Social safety in the working environment)* (2nd ed.). Kerckebosch. https://www.kerckebosch.nl/arbo-veiligheid/handleiding-sociale-veiligheidsindex-2832

Vranjes, I., Baillien, E., Vandebosch, H., Erreygers, S., & De Witte, H. (2018). When workplace bullying goes online: construction and validation of the Inventory of Cyberbullying Acts at Work (ICA-W). *European Journal of Work and Organizational Psychology*, *27*(1), 28–39. https://doi.org/10.1080/1359432X.2017.1363185

Waschgler, K., Ruiz-Hernández, J. A., Llor-Esteban, B., & Jiménez-Barbero, J. A. (2013). Vertical and Lateral Workplace Bullying in Nursing. *Journal of Interpersonal Violence*, *28*(12), 2389–2412. https://doi.org/10.1177/0886260513479027

Weatherbee, T. G. (2007). Cyberaggression in the workplace: Construct development, operationalization, and measurement (Dissertation at Saint

Continued SUPPLEMENTARY TABLE S1.

**INSTRUMENTS OF NWB CONSTRUCTS (**tests, questionnaires, scales) **STUDIES**	1. **GOAL** 2. **DIGITAL NWB:** Cyber enabled (CE) items[1] Cyber dependent (CD) items	**QUESTIONS ON:** 1. **ACTOR TYPE** 2. **ACTOR ROLE** **HARM**	**RATING SCALE: ANCHORS**	**DURATION**	**ITEMS**	**RELIABILITY/ CONSISTENCY**

Mary's University, Halifax). In *ProQuest Dissertations and Theses*. https://search.proquest.com/docview/304719031?accountid=14504%0Ahttp://godot.lib.sfu.ca/GODOT/hold_tab.cgi?url_ver=Z39.88-2004&rft_val_fmt=info:ofi/fmt:kev:mtx:dissertation&genre=dissertations+%26+theses&sid=ProQ:ProQuest+Dissertations+%26+Theses+A%26I&at

Weatherbee, T. G., & Kelloway, E. (2006). A case of cyberdeviancy: Cyberaggression in the workplace. In K. E. Kelloway, J. Barling, & J. . Hurrell (Eds.), *Handbook of Workplace Violence* (pp. 445–488). SAGE Publications, Inc. https://doi.org/10.4135/9781412976947.n19

Weiss, D., & Marmar, C. (1997). The Impact of Event Scale — Revised. In J. Wilson & T. Keane (Eds.), *Assessing psychological trauma and PTSD* (pp. 399–411). Guildford.

West, S. M., Crawford, K., & Whittaker, M. (2019). *Discriminationg systems: Gender, race, and power in AI.* https://ainowinstitute.org/discriminatingsystems.pdf

Xu, T., Magnusson Hanson, L. L., Lange, T., Starkopf, L., Westerlund, H., Madsen, I. E. H., Rugulies, R., Pentti, J., Stenholm, S., Vahtera, J., Hansen, Å. M., Kivimäki, M., & Rod, N. H. (2018). Workplace bullying and violence as risk factors for type 2 diabetes: a multicohort study and meta-analysis. *Diabetologia*, *61*(1), 75–83. https://doi.org/10.1007/s00125-017-4480-3

Zadro, L., Williams, K. D., & Richardson, R. (2004). How low can you go? Ostracism by a computer is sufficient to lower self-reported levels of belonging, control, self-esteem, and meaningful existence. *Journal of Experimental Social Psychology*, *40*(4), 560–567. https://doi.org/10.1016/j.jesp.2003.11.006

Zivnuska, S. L., Carlson, D. S., Carlson, J. R., Harris, K. J., Harris, R. B., & Valle, M. (2020). Information and communication technology incivility aggression in the workplace: Implications for work and family. *Information Processing & Management*, *57*(3), 102222. https://doi.org/10.1016/j.ipm.2020.102222
