# Supplementary material for: Beyond Bullying, Aggression, Discrimination, and Social Safety: Development of an Integrated Negative Work Behavior Questionnaire (INWBQ)
Source: Int J Environ Res Public Health. 2023 Aug 11;20(16):6564. doi: 10.3390/ijerph20166564 (PMC10454399; doi:10.3390/ijerph20166564)
Supplement: Supplementary file 1 [file ijerph-20-06564-s001.zip › Supplementary Table S4. INWBQ.pdf]

SUPPLEMENTARY TABLE S4. INWBQ

INWBQ Part I

The INWBQ measures your observation of physical, material, psychological, sociocultural, and digital forms of negative work behavior in your organization. Most people observing these behaviors choose how to act. Therefore, we also ask what you did.

I cordially invite you to participate in this study. The purpose of the study is twofold: to provide a scientifically sound instrument and to provide companies with tools to address this problem.

Completing the INWBQ-Part I on the Internet, which will take 10 to 15 minutes per module (physical, material, etc.). We will send you a module every day in the next five days. Your participation is voluntary, and the information obtained will be kept strictly confidential and anonymous.
All data from this survey will be stored in a secure and locked facility at the university.
If you would like more information about the study, or if you have any questions, please contact me by e-mail.

Part I NWB: item changes underscored, (source), ranking number.

How often did this happen last year at work?					What did you do? Select all that apply.				
☐ *Never*	☐ *Rarely*	☐ *At least once a month*	☐ *At least once a week*	☐ *Daily.*	☐ I *looked away*	☐ *I joined in*	☐ *I helped victims*	☐ *I suffered from this*	☐ *I did this*
					☐ *I walked away*	☐ *I laughed*	☐ *I assisted victims*	☐ *I tried to defend me*	☐ *I invited to do it*
					☐ *I shut my door*	☐ *I encouraged*	☐ *I defended victims.*	☐ *I was speechless.*	☐ *I acted this way.*
					☐ *I ignored*	☐ *I openly approved*	☐ *I openly disapproved it.*	☐ *I was bothered.*	☐ *Other*

Dimension Physical NWB (9 items: *6 overt, 3 covert*)

The following questions concern bodily types of negative work behavior in the past year. We first ask if you witnessed this behavior, and after that in what ways you participated in this situation.

What did you do in this situation?
Select all that apply:

How often did this happen last year at work?
Subcategory Bodily attacks (BA)

1. *Somebody showed their anger by breaking doors, windows, walls (HABS-U) 3.*
2. *Physical force was used against someone, it includes among others:*

☐ beating,
☐ kicking,
☐ slapping,

- ☐ stabbing,
- ☐ shooting,
- ☐ pushing,
- ☐ biting,
- ☐ pinching (Violence research) 2, 4.

3. Somebody was attacked on purpose with:

- ☐ an object
- ☐ a weapon (JVQ) 1.

Subcategory Bodily threats (BT)

4. *Not allowed drugs or alcohol were consumed on the job (I&O Deviance Scale) 2.*
5. *Threats of physical violence were made to somebody (LIPT) 1.*

Subcategory Body language (BL)

6. *Someone showed intimidating behaviors such as*

- ☐ finger-pointing,
- ☐ invasion of personal space,
- ☐ shoving,
- ☐ blocking the way (NAQ-R)1.

7. Someone was given dirty looks, or other negative eye-contact (BNGS) 2.

Subcategory Physical isolation (PI)

8. *Someone was isolated in a room far away from the others* (LIPT) 1.

Subcategory Physical tasks (PT)

9. Somebody had their key areas of responsibility removed or replaced with more trivial or unpleasant tasks (NAQ-R)1.

Dimension Material NWB (13 items: *3 overt,* 10 covert items)

The following questions concern material types of negative work behavior in the past year. We first ask if you witnessed this behavior, and after that in what ways you participated in this situation.

How often did this happen last year at work?

Subcategory Material Property (MP)

10. Somebody took items from someone's desk without prior permission. (UWBQ) 1.
11. *Someone damaged personal property or workplace property. (Cyber aggression) 2.*
12. Somebody falsified a receipt to get reimbursed for more money than spent on business expenses. (I&O Deviance scale) 3.
13. Somebody purposely dirtied someone's workplace. (CWB-C) 4.

Subcategory Material resources (MR)

14. Somebody withheld necessary information. (WBQ-BO) 1.
15. Somebody shared confidential information. (FSS) 2.
16. Someone took credit for others work or ideas. (GWHQ) 3.

Subcategory Material Job decisions (MJ)

17. Job decisions are made on inaccurate and incomplete information. (Injustice scale)1.
18. For some people the level of pay was unfair. (Injustice scale) 2.

Subcategory Material sabotage (MS)

19. Somebody purposely came late to an appointment or meeting. (CWB-C) 1.
20. Somebody refused to provide needed sources or equipment. (BNGS) 2.
21. *Somebody neglected to follow their boss's instructions (I&O Deviance scale) 3.*

Subcategory Material displays (MD)

22. *Someone distributed dirty pictures or stories. (SEQ) 1.*

Dimension Psychological NWB (14 items: *6 overt*, 8 covert items)

The following questions concern psychological types of negative work behavior in the past year. We first ask if you witnessed this behavior, and after that in what ways you participated in this situation.

How often did this happen last year at work?

Subcategory Psychological Verbal (PV)

23. *Someone was humiliated or belittled personally in front of others? (GHWQ) 1.*
24. Somebody's correct decisions and achievements have been treated with disdain (EAPA-T-R) 2.
25. Some co-workers spread false rumors about someone (HABS-CS) 3.
26. *Somebody got persistent criticism about the errors or mistakes he/she made (NAQ-R) 4.*
27. Somebody was unfairly blamed for work problems (WCM) 6.
28. *Someone was yelled at and loudly scolded (LIPT) 8.*

Subcategory Psychological Nonverbal (PN)

29. A mean prank was played on someone at work (I&O Deviance Scale) 1.
30. Somebody was intentionally given no work or assignments below their job description (FSS) 2.

Subcategory Psychological Threats (PT)

31. *Oral and written threats were made to people (LIPT) 1.*
32. Someone received unreasonable work demands (WCM) 2.

Subcategory Psychological Excluding (PE)

33. Somebody at work was treated as if he/she weren't there (WOS) 1.
34. Somebody intentionally ignored another person/people (IAS-T) 2.

Subcategory Psychological Hinder work (PH)

35. *Somebody was constantly interrupted (LIPT) 1.*
36. *Some co-workers refused to help someone at work (CWB-C) 2.*

Dimension Sociocultural NWB (15 items: 5 *overt,* 10 covert items)

The following questions concern types of negative work behavior on specific social characteristics, such as sex or race, in the past year. We first ask if you witnessed this behavior, and after that in what ways you participated in this situation.

How often did this happen last year at work?

Subcategory Sociocultural Identity (SI)

37. *Somebody made sexist jokes in your presence (SEQ) 1.*
38. *Somebody used racial slurs to describe workers (FSS) 2.*
39. Somebody was not given needed information to do their job because of ethnicity (EHE) 3.
40. Somebody got insulting comments about their private life (WHS) 4.
41. Negative comments were made to somebody about their personality (GHWQ) 4.
42. *Somebody's handicap was ridiculed (LIPT) 5.*
43. People gossiped about the way someone moves or talks (Mobbing scale) 6.
44. Somebody was discriminated on their age? (SVI) 7.

Subcategory Sociocultural Demographic (SD)

45. *Someone was criticized for not behaving ''like a woman should'' (SEQ) 1.*
46. *Religious remarks were made at someone (I&O Deviance Scale) 2.*
47. Somebody experienced threatening conduct based on their national origin (Violence research) 3.
48. Somebody experienced conduct based on their birth or other status (Violence research) 4.

Subcategory Sociocultural Organization (SO)

49. Somebody showed unwanted conduct on union trade members. (Violence research) 1.
50. There is negative conduct between people of different departments (Monitor social safety) 1.
51. There has always been an influential group in this department that no one ever crosses (POPS)1.

Dimension Digital NWB (18 items: 2 digital use, *5 overt,* 11covert/ anonymous)

Subcategory Viral reach (VR)

52. Which technology and tools are used to work with? (Digital safety)

Select all that apply:

- ☐ Desktop computer
- ☐ Laptops/Tablets

- ☐ Mobile Phones
- ☐ Video/Audio recording devices
- ☐ E-mail service
- ☐ Collaborative tools (e.g., Google Docs)
- ☐ Cloud storage (e.g., Dropbox)
- ☐ USB devices
- ☐ General websites, including search engines.
- ☐ Networking platform (e.g., Facebook, LinkedIn, Weibo, Twitter, etc.)
- ☐ GPS

Subcategory Digital duration (DD)

53. During the last 12 months, how much of the time a day have you spent digital activities? Please estimate:

- ☐ 1/2-1 hour,
- ☐ 1-2 hours,
- ☐ over 2 hours

The following questions concern digital forms of negative work behavior in the past year. We first ask if you witnessed this behavior, and after that in what ways you participated in this situation.

How often did this happen last year at work?

Subcategory Cyber enabled (CE)

54. *Somebody used capital letters to shout through e-mail (Cyber incivility) 1.*
55. *Somebody copied extracts from messages so that the meaning of the original message was distorted (NAQ-R) 2.*
56. Somebody received messages containing false information about themselves (WCM) 2.
57. *Somebody shared photos or videos on the internet to make fun of others (ICA-W)3.*
58. *Somebody was excluded from the social community online (e.g., Facebook, Twitter) (CBQ) 4.*
59. Somebody was insulted, threatened, or intimidated by means of ICTs (ICA-W) 5.
60. Somebody discussed negative feelings towards clients, customers, or coworkers on social media (WSMQ) 6.
61. *Persistent criticism of someone's work or performance has been made on digital media (CBQ) 7.*

Subcategory Cyber dependent (CD)

62. Somebody's personal information was hacked to harm this person (ICA-W) 1.
63. Someone's data were stolen, including data stored in the cloud (Digital safety) 1.
64. Somebody pretended to be another online, without permission. (cyber-stalking victimization) 1.
65. Somebody's computer identity has been hijacked (CBQ) 1.

66. Somebody's e-mails were intercepted (Digital safety) 1.
67. A disinformation campaign was used against someone (Digital safety) 2.
68. Viruses were intentionally sent to someone's email address (CBQ) 2.
69. Someone's online activities have been surveilled (Digital safety) 3.

Occurrence patterns NWB (7 items)

The following questions ask if you could observe a particular pattern in the negative behavior.

Subcategory Escalation (Esc)

70. Did negative behavior escalate from mild into more serious or physical natures over time? (self-formulated)
71. Did the digital behavior escalate from virtual into the real world? (cyberstalking)
72. Did negative work behavior get worse because no one reported it? (CWB-C)
73. Did someone ask persons to stop to prevent further escalation? (self-formulated)

Subcategory Visibility (Vis)

74. Did the negative behavior change from covert into a more overt and visible nature (self-formulated)
75. Was the negative behavior invisible before somebody noticed it? (self-formulated)
76. Do you know who might be behind these types of attacks? (Digital safety)

INWBQ part II

Sometimes individuals who experience negative work behaviors may have health complaints, feelings of anger or anxiety. Employees may also call in sick or resign. With 11 questions in multiple choice style, we ask you about what consequences you noticed of negative work behavior.

I cordially invite you to participate in this study. The purpose of INWBQ-Part II Harm is to help organizations understand the relationship between the risk and its damage, enabling effective risk management.

Completing the INWBQ -Part II on the Internet, which will take 15-20 minutes. Your participation is voluntary, and the information obtained will be kept strictly confidential and anonymous.
All data from this survey will be stored in a secure and locked facility at the university.
If you would like more information about the study, or if you have any questions, please contact me by e-mail.

Part II HARM

The following questions ask about the inflicted harm to you, other people, or your organization by the different types of negative work behavior (NWB) as mentioned in the questions before.

**How often did you observe this last year at work?**				
☐ *Never*	☐ *Rarely*	☐ *At least once a month*	☐ *At least once a week*	☐ *Daily.*

Dimension Bodily harm (3 items/ 10 multiple choice options)

How often did you observe this last year at work?

Subcategory Bodily injury (BI)

1. Was somebody physical damaged by incidents of NWB at work? (SVI) 1.

Subcategory Psychosomatic complaints (PC)

2. **As far as you know, did workers experience one of the following health complaints because of the NWB: Select all that apply:**

☐ headache (SVI) 1.

☐ musculoskeletal disorders i.e., stiffness of the neck or shoulders, lumbago, and pain of two or more joints [1] 1.

☐ poor general health (LIPT) 1. [2–4] 2.

☐ a bad cold or flu (Cyber aggression) 2.

☐ sleeping problems (Cyber aggression) 2, [5, 6] 3.

☐ nausea or an upset stomach (SVI) 3.

☐ chest pain (FSS) 4.

Subcategory Illness (Il)

3. **As far as you know, did workers have a diagnosis of the following physical health problems (LIPT): Select all that apply:**

☐ diabetes type 2 (Xu et al., 2018) 4.

☐ change in blood pressure [5] 4.

☐ cardiovascular disease [7–9] 2.

Dimension Material damage (4 items/ 16 multiple choice options)

How often did you observe this last year at work?

Subcategory Material personal damage (MD)

4. **As far as you know, what damage incidents of NWB caused to workers?**
   Select all that apply:

- ☐ general damage causing financial costs (LIPT) 1.
- ☐ stolen data (Digital safety) 1.
- ☐ declined performance (NAQ-R) 2; (e.g., due to memory problems, less creative ideas, worsened verbal tasks) [8, 10] 1.
- ☐ intention to leave the job [8, 10, 11] 1.
- ☐ lost working time (because of worrying, avoiding) [10] 3.

Subcategory Material organizational damage (OD)

5. **As far as you know, did the NWB inflict one of the following material damages to your organization?**
   Select all that apply:

- ☐ reduced productivity [8] 1.
- ☐ increased job turnover [8, 10, 11] 2.
- ☐ sickness absence [2, 4, 12] 4.

6. **As far as you know, did the NWB inflict one of the following long term organizational material damages?**
   Select all that apply:

- ☐ replacement costs by employee turnover [8, 10, 11] 2.
- ☐ extra costs for consultants and interventions [8, 10] 3.
- ☐ reputational damage (Citron & Franks, 2014; Porath & Pearson, 2010) 4.
- ☐ reduced commitment of workers to each other [10] 5.
- ☐ reduced commitment of workers to the organization [10] 5.

Subcategory Material societal damage (SD)

7. **As far as you know, did the NWB inflict one of the following *societal* costs?**

- ☐ additional medical treatment [8, 14] 5.
- ☐ premature retirement [8] 5.
- ☐ disability retirement [15] 5.
- ☐ additional costs to the criminal justice system [8] 6.

Dimension Mental harm (3 items/ 13 multiple choice options)

How often did you observe this last year at work?

Subcategory Temporarily mental complaints (TC)

8. **As far as you know, did the NWB inflict one of the following temporarily complaints on workers? Select all that apply:**

- ☐ felt anxious (Cyber aggression) 1.
- ☐ felt embarrassed (WCM) 2.

- ☐ felt stressed out (FSS) 2.
- ☐ felt angry (Cyber aggression) 3.
- ☐ affect social contacts (LIPT) 4.
- ☐ had difficulties concentrating (Cyber harassment) 5.

Subcategory Persistent mental complaints (PC)

9. As far as you know, did the NWB inflict the following persistent complaints on workers? Select all that apply:

- ☐ effects self-esteem (LIPT) 1.
- ☐ felt bad and unhappy (JOS) 2.
- ☐ felt depressed (FSS) 2.
- ☐ am unsatisfied with my job (Cyber aggression) 3.

10. Looking at yourself, did the NWB inflict the following combinations of complaints? Select all that apply:

- ☐ felt a combination of negative mood [5, 16, 17]1; loss of interest (SVI) 6, withdrawal (Slitter 2012) 3; feeling worthless (Cyber aggression) 1, feeling embarrassed (GWHQ) 2; and sleep problems (WCM) 3. (Depressive symptoms, Hansen et al., 2014; Magnavita et al., 2019; Verkuil et al., 2015)
- ☐ felt a combination of flashbacks (SVI) 1, avoid places-people-activities (FSS) 2, dreams and images of past events (Cyber aggression) 7, blame myself (Cyber harassment) 2, emotional reactions as anger (Cyber aggression) 3, (PTSD symptoms, [16, 19])
- ☐ felt a combination of emotional exhaustion [4, 16] 2; changed work attitudes (e.g., cynic, less professional) [8, 10] 1; little self-esteem (JOS) 1; I am not the one I used to be (SVI) 7. (Burnout symptoms, Savicki et al., 2003; Sliter et al., 2012; Smoktunowicz et al., 2015)

Dimension Social harm (after Fredericksen & McCorkle, 2013) (1 item/ 10 multiple choice)

How often did you observe this last year at work?

Subcategory Social harm (SH)

11. As far as you know, did the NWB inflict the following social consequences for workers? Select all that apply:

- ☐ affected family members of workers [17, 24, 25]1.
- ☐ caused damage at home (LIPT) 1.
- ☐ affected the possibilities to communicate with colleagues (Mobbing with effects) 1.
- ☐ affected the dignity of woman at work (Violence research) 2.
- ☐ affected the ethnic identity of workers (EHE) 2
- ☐ invasion of workers' privacy [13] 2.
- ☐ affected friend networks (Digital safety) 2.

- ☐ additional medical treatment [14] 2.
- ☐ premature retirement [8] 2.
- ☐ disability retirement [15] 2.

Table 6. Integrated Negative Work Behavior Questionnaire, INWBQ Part I NWB and Part II Harm: Dimensions, Subcategories, Items (covert and overt), Item source, Ranking. Note: behind each item the item source, and the ranking number of this NWB and harm frequency in subcategory of dimension.

References Supplementary Table S4:

[1] Takaki, J.; Taniguchi, T.; Hirokawa, K. Associations of Workplace Bullying and Harassment with Pain. *Int. J. Environ. Res. Public Health*, **2013**, *10* (10), 4560–4570. https://doi.org/10.3390/ijerph10104560.

[2] Elovainio, M.; Kivimaki, M.; Vahtera, J. Organizational Justice: Evidence of a New Psychosocial Predictor of Health. *Am. J. Public Health*, **2002**, *92*, 105–108.

[3] Einarsen, S.; Raknes, B. I.; Matthiesen, S. B.; Hellesøy, O. H. Helsemessige Aspekter Ved Mobbing i Arbeidslivet: Modererende Effekter Av Sosial Støtte Og Personlighet. *Nord. Psykol.*, **1996**, *48* (2), 116–137. https://doi.org/10.1080/00291463.1996.11863874.

[4] Hyde, M.; Jappinen, P.; Theorell, T.; Oxenstierna, G. Workplace Conflict Resolution and the Health of Employees in the Swedish and Finnish Units of an Industrial Company. *Soc. Sci. Med.*, **2006**, *63* (8), 2218–2227. https://doi.org/10.1016/j.socscimed.2006.05.002.

[5] Thurston, R. C.; Chang, Y.; Matthews, K. A.; von Känel, R.; Koenen, K. Association of Sexual Harassment and Sexual Assault with Midlife Women's Mental and Physical Health. *JAMA Intern. Med.*, **2019**, *179* (1), 48. https://doi.org/10.1001/jamainternmed.2018.4886.

[6] Hansen, Å. M.; Hogh, A.; Garde, A. H.; Persson, R. Workplace Bullying and Sleep Difficulties: A 2-Year Follow-up Study. *Int. Arch. Occup. Environ. Health*, **2014**, *87* (3), 285–294. https://doi.org/10.1007/s00420-013-0860-2.

[7] Jacob, L.; Kostev, K. Conflicts at Work Are Associated with a Higher Risk of Cardiovascular Disease. *GMS Ger. Med. Sci.*, **2017**, *15*, 1–8. https://doi.org/10.3205/000249.

[8] Giga, S. I.; Hoel, H.; Lewis, D. *The Costs of Workplace Bullying*; Department for Business, Enterprise and Regulatory Reform., 2008.

[9] Kivimaki, M.; Virtanen, M.; Vartia, M.; Elovainio, M.; Vahtera, J.; Keltikangas-Järvinen, L. Workplace Bullying and the Risk of Cardiovascular Disease and Depression. *Occup. Environ. Med.*, **2003**, *60*, 779–783.

[10] Porath, C. L.; Pearson, C. M. The Cost of Bad Behavior. *Organ. Dyn.*, **2010**, *39* (1), 64–71. https://doi.org/10.1016/j.orgdyn.2009.10.006.

[11] Namie, G. *How Employers Pay for Bullying*; 2008.

[12] Slany, C.; Schütte, S.; Chastang, J.-F.; Parent-Thirion, A.; Vermeylen, G.; Niedhammer, I. Psychosocial Work Factors and Long Sickness Absence in Europe. *Int. J. Occup. Environ. Health*, **2014**, *20* (1), 16–25. https://doi.org/10.1179/2049396713Y.0000000048.

[13] Citron, D. K.; Franks, M. A. Criminalizing Revenge Porn. *Wake For. Law Rev. 345*, **2014**, No. 2014.

[14] Sabbath, E. L.; Williams, J. A. R.; Boden, L. I.; Tempesti, T.; Wagner, G. R.; Hopcia, K.; Hashimoto, D.; Sorensen, G. Mental Health Expenditures: Association with Workplace Incivility and Bullying among Hospital Patient Care Workers. *J. Occup. Environ. Med.*, **2018**, *60* (8), 737–742. https://doi.org/10.1097/JOM.0000000000001322.

[15] Nielsen, M. .; Gjerstad, J.; Jacobsen, D. P.; Einarsen, S. V. Does Ability to Defend Moderate the Association between Exposure to Bullying and Symptoms of Anxiety? *Front. Psychol.*, **2017**, *8* (NOV), 1–11. https://doi.org/10.3389/fpsyg.2017.01953.

[16] Verkuil, B.; Atasayi, S.; Molendijk, M. L. Workplace Bullying and Mental Health : A Meta- Analysis on Cross-Sectional and Longitudinal Data. *PLoS One*, **2015**, *10* (8), 1–17. https://doi.org/10.1371/journal. pone0135225.

[17] Zivnuska, S. L.; Carlson, D. S.; Carlson, J. R.; Harris, K. J.; Harris, R. B.; Valle, M. Information and Communication Technology Incivility Aggression in the Workplace: Implications for Work and Family. *Inf. Process. Manag.*, **2020**, *57* (3), 102222. https://doi.org/10.1016/j.ipm.2020.102222.

[18] Magnavita, N.; Di Stasio, E.; Capitanelli, I.; Lops, E. A.; Chirico, F.; Garbarino, S. Sleep Problems and Workplace Violence: A Systematic Review and Meta-Analysis. *Front. Neurosci.*, **2019**, *13* (October). https://doi.org/10.3389/fnins.2019.00997.

[19] Baran Tatar, Z.; Yuksel, S. Mobbing at Workplace - Psychological Trauma and Documentation of Psychiatric Symptoms. *Arch. Neuropsychiatry*, **2018**, *56* (1), 57–62. https://doi.org/10.29399/npa.22924.

[20] Savicki, V.; Cooley, E.; Gjesvold, J. Harassment as a Predictor of Job Burnout in Correctional Officers. *Crim. Justice Behav.*, **2003**, *30* (5), 602–619. https://doi.org/10.1177/0093854803254494.

[21] Smoktunowicz, E.; Baka, L.; Cieslak, R.; Nichols, C. F.; Benight, C. C.; Luszczynska, A. Explaining Counterproductive Work Behaviors among Police Officers: The Indirect Effects of Job Demands Are Mediated by Job Burnout and Moderated by Job Control and Social Support. *Hum. Perform.*, **2015**, *28* (4), 332–350. https://doi.org/10.1080/08959285.2015.1021045.

[22] Sliter, K. A.; Sliter, M. T.; Withrow, S. A.; Jex, S. M. Employee Adiposity and Incivility: Establishing a Link and Identifying Demographic Moderators and Negative Consequences. *J. Occup. Health Psychol.*, **2012**, *17* (4), 409–424. https://doi.org/10.1037/a0029862.

[23] Fredericksen, E. D.; McCorkle, S. Explaining Organizational Responses to Workplace Aggression. *Public Pers. Manage.*, **2013**, *42* (2), 223–238. https://doi.org/10.1177/0091026013487050.

[24] Hoobler, J. M.; Brass, D. J. Abusive Supervision and Family Undermining as Displaced Aggression. *J. Appl. Psychol.*, **2006**, *91* (5), 1125–1133. https://doi.org/10.1037/0021-9010.91.5.1125.

[25] Thompson, M. J.; Carlson, D. S.; Kacmar, K. M.; Vogel, R. M. The Cost of Being Ignored: Emotional Exhaustion in the Work and Family Domains. *J. Appl. Psychol.*, **2020**, *105* (2), 186–195. https://doi.org/10.1037/apl0000433.
